# Regulation of platelet-activating factor-induced interleukin-8 expression by protein tyrosine phosphatase 1B

**DOI:** 10.1186/s12964-019-0334-6

**Published:** 2019-03-04

**Authors:** Geneviève Hamel-Côté, Fanny Lapointe, Daniel Gendron, Marek Rola-Pleszczynski, Jana Stankova

**Affiliations:** 1 Immunology Division, Department of Pediatrics, Faculty of Medicine and Health Sciences, Université de Sherbrooke, Sherbrooke, QC J1H 4N5 Canada; 2 Agriculture and Agri-Food Canada, Dairy and Swine Research and Development Center, 2000 College Street, Sherbrooke, QC Canada

**Keywords:** PTP1B, Platelet-activating factor, Interleukin-8, GSK-3, CCAAT-enhancer-binding protein(C/EBP)

## Abstract

**Background:**

Platelet-activating factor (PAF) is a potent lipid mediator whose involvement in the onset and progression of atherosclerosis is mediated by, among others, the modulation of cytokine expression patterns. The presence of multiple potential protein-tyrosine phosphatase (PTP) 1B substrates in PAF receptor signaling pathways brought us to investigate its involvement in PAF-induced cytokine expression in monocyte-derived dendritic cells (Mo-DCs) and to study the pathways involved in this modulation.

**Methods:**

We used in-vitro-matured human dendritic cells and the HEK-293 cell line in our studies. PTP1B inhibition was though siRNAs and a selective inhibitor. Cytokine expression was studied with RT-PCR, luciferase assays and ELISA. Phosphorylation status of kinases and transcription factors was studied with western blotting.

**Results:**

Here, we report that PTP1B was involved in the modulation of cytokine expression in PAF-stimulated Mo-DCs. A study of the down-regulation of PAF-induced IL-8 expression, by PTP1B, showed modulation of PAF-induced transactivation of the IL-8 promoter which was dependent on the presence of the C/EBPß -binding site. Results also suggested that PTP1B decreased PAF-induced IL-8 production by a glycogen synthase kinase (GSK)-3-dependent pathway via activation of the Src family kinases (SFK). These kinases activated an unidentified pathway at early stimulation times and the PI3K/Akt signaling pathway in a later phase. This change in GSK-3 activity decreased the C/EBPß phosphorylation levels of the threonine 235, a residue whose phosphorylation is known to increase C/EBPß transactivation potential, and consequently modified IL-8 expression.

**Conclusion:**

The negative regulation of GSK-3 activity by PTP1B and the consequent decrease in phosphorylation of the C/EBPß transactivation domain could be an important negative feedback loop by which cells control their cytokine production after PAF stimulation.

**Electronic supplementary material:**

The online version of this article (10.1186/s12964-019-0334-6) contains supplementary material, which is available to authorized users.

## Plain English summary

Atherosclerosis is an inflammatory disease affecting the wall of large and medium-sized arteries. In risk areas, the wall of blood vessels is under constant reconstruction, resulting in a low-grade inflammatory state, facilitating lipid deposits and the recruitment of immune cells such as monocytes. These monocytes can differentiate into immature dendritic cells which are responsive to inflammatory molecules such as platelet-activating factor. This lipid is one of the first mediators produced by endothelial cells activated by lipid deposits. PAF-activated immature dendritic cells can orchestrate the progression of an inflammatory state through the production of pro- or anti-inflammatory mediators such as cytokines depending on how they integrate the different signals coming from their environment. Here we show that the protein tyrosine phosphatase PTP1B could be an important integration point since decreasing its activity can change the cytokine pattern induced by PAF through the modulation of specific signaling pathways.

## Background

Atherosclerosis is the underlying cause of many cardiovascular diseases and is a widespread chronic condition affecting large and medium-size arteries. Lipid accumulation and modifications in the arterial wall may act as the triggering event of the inflammatory condition, where the activated endothelium, among others, increases its adhesion molecule expression and secretes chemokines and cytokines leading to the recruitment of circulatory monocytes. These will enter the intima and differentiate into macrophages or monocyte-derived dendritic cells (Mo-DCs), according to the composition of the environment, thus increasing the dendritic cell (DC) population, which is also composed of DCs differentiated from committed dendritic cell precursors [1, 2]. While the involvement of macrophages in atherosclerotic progression is well characterized, less is known about the contribution of DCs and Mo-DCs.

The latter form a subtype of sensing myeloid cells able to produce a wide range of cytokines and chemokines. They fine-tune the progression of atherosclerosis by secreting, among others, cytokines that decrease the pro-inflammatory content of the plaque or that contribute to stabilize it, such as IL-10 and Transforming Growth Factor beta (TGFβ), known to attenuate lymphocyte proliferation and expression of pro-inflammatory genes [2–9]. However, Mo-DCs can also contribute to plaque destabilization by secreting Tumour-Necrosis Factor α (TNFα) [10], which is involved in matrix metalloproteases (MMP) expression and leukocyte adhesion [3, 11]. They also secrete interleukin (IL)-6, known for its involvement in lipid homeostasis, for its modulation of adhesion molecules and cytokine expression and whose systemic levels are correlated with plaque development in humans [11–17]. These cells also regulate the composition of their environment by recruiting other cells via chemokine production such as CCL2, involved in monocyte recruitment [18], and IL-8 [13, 18] whose levels are increased in human plaques after strokes and transient ischemic accidents [19]. IL-8 is particularly interesting given its involvement in many cellular responses modulating atherogenic progression. For instance, IL-8 increases endothelial and vascular smooth muscle cell (SMC) migration, retraction/contraction and proliferation [20–23]. It facilitates monocyte recruitment by inducing chemotaxis, alone or in synergy with CCL2, and by increasing firm adhesion to endothelial cells [24–26]. Moreover, IL-8 and its murine homolog KC contribute to plaque destabilization n by increasing matrix metalloproteinase (MMP)-2 and MMP-9 expression [23, 27].

Among stimulating factors encountered by Mo-DCs in the atherosclerotic plaque, one of the earliest cellular products found, platelet-activating factor (PAF) [28] is known to induce a wide range of cytokine expression involved in atherosclerosis. For example, TGFß and IL-10 production is activated in macrophages and DCs [4, 9], IL-6 in vascular SMC [29], and TNFα and IL-8 in human monocytes [30, 31]. This wide range of cytokines produced could be due to the fact that, by activating its receptor (PAFR), a G-protein coupled receptor (GPCR), PAF modulates several different signaling pathways. Among others, the NFκB pathway seems to be important given that PAF increases the expression of many cytokines such as IL-8, TNFα and CCL2 in different cells types [32–36].

Besides the NFκB pathway, however, little is known about the signaling pathways involved in PAF-induced IL-8 expression. Studies with other stimuli show that the dimeric transcription factor AP-1 (Activator protein-1) can be involved in IL-8 expression, as shown by Ryoo and colleagues in LDL-stimulated SMC [37]. PAF can rapidly induce the expression of the AP-1 subunit c-FOS [38] and the phosphorylation on the activating sites (Ser63 and Ser73) of c-Jun in fibroblasts [39] and in human colon carcinoma cells [40]. On the other hand, it does not induce the phosphorylation on the activating sites (Thr 183 and Tyr 185) of JNK (c-Jun NH2-terminal kinases) in many cell types such as neutrophils, SMC and epidermal cells over-expressing the PAFR, suggesting that PAF-induced AP-1 trans-activation is a cell type-dependent event [41–44].

Signal transducer and activator of transcription (STAT)3 and C/EBPß have also been shown to be involved in the modulation of IL-8 expression by various stimuli [45–47]. Whereas PAF trans-activates STAT3 in a Tyk2/Jak2- and Src-dependent manner [48–50], PAF-induced C/EBPß trans-activation is less well characterized. However, in rat small intestinal cells, PAF can increase the binding of nuclear complexes consisting of C/EBPß and the p65 and p50 NFκB subunits to a C/EBP consensus oligonucleotide probe ( [51]. C/EBPß belongs to the CCAAT-enhancer-binding protein family, a basic region leucine zipper (bZIP) subfamily [52]. C/EBPß is composed of three isoforms, generated by alternative translation initiation sites, all containing the DNA-binding domain [47]. Using cells with mutations in alternative translation initiation sites, it has been suggested that the full-length isoform, C/EBPß-1 or LAP-1 (Liver-enriched activator protein) would be involved in senescence via the modulation of IL-6 and IL-8 expression, whereas C/EBPß-2 or LAP-2 would be involved in cellular proliferation [47]. The third isoform, C/EBPß-3 or LIP (Liver-enriched inhibitory protein) lacks the transactivation domain (TAD) and regulates promoter activity by occupying and blocking DNA sites or by modulating transcription factor complexes bound to the consensus sites [47, 53]. All C/EBPß isoforms are modulated by phosphorylation by different kinases such as ERK, p38MAPK, glycogen synthase kinase (GSK)-3 and PKC which can modulate C/EBPß cellular localization, DNA binding, and transactivation capacities, depending on the cell type, DNA sequence, the stimulus or the isoform involved [52, 54–56].

Little is known about PAF-induced Mo-DCs cellular responses, but among possible modulators of the PAF-induced signaling pathways involved in cytokine production, the protein tyrosine phosphatase PTP1B is of interest as it modulates several kinases and some transcription factors mentioned above such as Jak2, Tyk2, Src or the MAP kinase p38 [48, 56–62]. Moreover, we have previously shown that PAF activates PTP1B, which is involved in PAFR signal transduction leading to the production of IL-6, an atherogenic cytokine [63]. Therefore, we investigated the involvement of this PTP in PAF-induced signal transduction and its impact in PAF-induced cytokine expression patterns, focusing on IL-8 expression in Mo-DCs. Since immature Mo-DCs (iMo-DCs) can be found early in the atherosclerosis plaque, investigating the effects of PTP1B on PAF-induced IL-8 expression and the underlying signaling pathways in these cells, may be relevant to understanding PAF-triggered mechanisms modulating disease progression.

## Methods

### Reagents and chemical products

PAF (C-16), wortmannin, MK-2206 and SB 216763 were from Cayman Chemicals (Ann Arbor, MI, USA). PTP1B inhibitor was from Calbiochem (San Diego, CA, USA). PP2 was from Alexis, purchased from Cedarlane (Burlington, ON, Canada). BSA for cell culture, cOmplete, MiniProtease Inhibitor Tablets and ATP for luciferase assays were from Roche Diagnostics (Laval, QC, Canada). Dulbecco’s Modified Eagle Medium (DMEM, high glucose) and D-Luciferin Na^+^ Salt were from Invitrogen (Burlington, ON, Canada). Fetal bovine serum (FBS) was from PAA Laboratories (Etobicoke, ON, Canada). TransIT-LT1 transfection reagent was from Mirius Bio (Medicorp Montréal, QC, Canada). Puromycin was from Wisent. Syber Green was from Molecular Probes, purchased from Cedarlane (Burlington, ON, Canada). ELISA MAX DELUXE set IL-8 was from Biolegend (San Diego, CA, USA).

Donkey anti-goat IgG HRP, mouse anti-pTyr204-ERK, and rabbit anti-ERK antibodies were from Santa-Cruz (LaJolla, CA, USA). Rabbit anti-phospho-Thr180/Tyr182-p38, rabbit anti-p38, rabbit anti-IκBα, rabbit anti-GSK-3ß, rabbit anti-Ser21/9 GSK-3, rabbit anti-pTyr1068 EGFR, rabbit anti-EGFR anti-rabbit HRP and anti-mouse HRP antibodies were from Cell Signaling (New England Biolabs Ltd., Pickering, ON, Canada). Mouse anti-vinculin and anti-FLAG antibodies, paraformaldehyde (PFA), leupeptin, pepstatin and BSA essentially IgG-free were from Sigma-Aldrich, (Oakville, ON, Canada). Amersham ECL Select Western Blotting Detection Reagent and nitrocellulose membranes (Hybond ECL) were from GE (GE Heathcare, Piscataway, NJ, USA). RhGM-CSF and rhIL-4 were from Peprotech (Rocky Hill, NJ, USA). Mouse anti-CD1a-FICT or -Phycoerythrin (PE), purified Anti-human IL-8, mouse anti-human CD86 purified IgG1κ, anti-human CD83-PE, −FITC conjugated, mouse Isotype Control IgG1κ, FITC Rat IgG1κ Isotype Control, mouse isotype IgG1κ control antibodies and BD GolgiStop were from BD Pharmingen. Goat anti-mouse IgG-Cy5 antibody was from Jackson Immunoresearch, purchased from Cederlane (Burlington, ON, Canada).

### siRNA sequences

We used a mix of 3 siRNA duplexes that we designed using siRNA Wizard Guideline (http://www.sirnawizard.com) and Ambion website. First one is against the PTP1B mRNA region 338–357: (5′-AUAGGUACAGAGACGUCAGUU-3′). The second is against region 702–720: (5′-CCAAGAAACUCGAGAGAUC-3′) and the last one, against the region 2038–2056 (5′-AUCCUCAGGUAGUACUGGGUU-3′). SiRNAs duplexes were from Sigma-Aldrich, with a UU overhang. SiRNAs controls (mission siRNA Universal Negative control #1) were from Sigma-Aldrich (Oakville, ON, Canada).

### Cell culture

HEK-293 (CRL. 1573, American Type Culture Collection (ATCC), Rockville, USA) stably transfected with pIRES_puro_PAFR_HA (HEK-PAFR) were grown in DMEM, 5% FBS at 37 °C 5% CO_2_ in a humidified atmosphere with puromycin, 5 μg/ml, penicillin, 60 μg/ml and streptomycin, 100 μg/ml.

Monocyte-derived dendritic cells (Mo-DCs) were generated from monocytes obtained from healthy volunteers after informed consent in accordance with protocol number 93–04 approved by the Comité d’Éthique de la recherche chez l’Humain adhering to the Declaration of Helsinki principles, Université de Sherbrooke. Monocytes were isolated from peripheral venous blood after enrichment by dextran sedimentation followed by purification by density gradient centrifugation on Ficoll-Hypaque and after adhesion on autologous serum-coated Petri dishes. Following intensive washings, adherent cells were recovered and cultured for 7 days in RPMI 1640 supplemented with 10% FBS, rhGM-CSF (20 ng/ml) and rhIL-4 (20 ng/ml) at 1.6 × 10^6^ cells/ml in a 6-well plate, 3 ml/well. Medium was changed after 24-48 h by removing half of the volume and adding the same volume of new medium. On day 5, the medium was changed by removing 1.5 ml, but refilling with medium containing only 10 ng/ml of each cytokine. Finally, at the end of day 6, 3 ml of RPMI 5% FBS without any cytokines was added to begin the starvation of the cells. On day 7, cells were collected and stimulated as described below. Aliquots of cells were routinely kept for determining, by flow cytometry, their differentiation and maturation state by staining with anti-CD83 or anti-CD86 antibodies, experiments where CD83 levels were too high were discarded.

Where mentioned, cells were transfected with siRNAs: on day 4, a solution of 2 μM siRNA in RPMI 1640 (75 μl) containing 2.3 μl TransiT LT was added to 4.8 × 10^6^ cells (1 well of a 6-well plate, 1.5 m m) and incubated for 6 h at 37 °C, 5% CO_2_. After that, 1.5 ml of fresh medium with 20 ng/ml of each cytokine and 10% FBS was added. A second transfection of siRNAs was done 30 h after the first one: 1.53 μM siRNA in RPMI 1640 (75 μl) containing 2.3 μl TransIT LT was added to 4.8 × 10^6^ cells (1 well of a 6-well plate, 1.5 ml) and incubated for 6 h at 37 °C, 5% CO_2_. After that, 1.5 ml of fresh medium with 10 ng/ml of each cytokine and 10% FBS was added.

RNA Isolation and Real-Time Semi-Quantitative PCR: Mo-DCs were collected on day 7, counted and incubated at 10^6^ cells/ml in RPMI+ 0.2% BSA for 3 h with agitation every 30–45 min. Next, cells were stimulated with different PAF concentrations for 5 h, then, RNA was obtained using Trizol reagent (Invitrogen, Burlington, ON, Canada) according to the manufacturer’s instructions. After quantification, 1.0 μg of RNA was converted to cDNA, QuantiTect Reverse Transcription Kit, according to the manufacturer’s instruction. GAPDH, RPL13a, IL-6, TNFα, IL-8, and PTP1B expression was measured using real-time PCR performed with Syber Green 1 on a Rotor-Gene 3000 (Corbett Research, Kirkland, QC, Canada) as described previously [12]. The following oligonucleotide primer sets were obtained from IDT (Coralville, Ind, USA):

humanGAPDH:

Fwd, 5′-GATGACATCAAGAAGGTGGTGAA-3′ Rvs, 5′-GTCTTACTCCTTGGAGGCCATGT-3′ human RPL13a:

Fwd, 5′-GTGCGTCTGAAGCCTACAAG-3′ Rvs, 5′-TCTTCTCCACGTTCTTCTCG-3′ human IL-8:

Fwd, 5′-TTCTGCAGCTCTGTGTGAAG-3′ Rvs, 5′-AAACTTCTCCACAACCCTCTG-3′ human PTP1B:

Fwd, 5’ACAGAGTGATGGAGAAAGGTTC-3′ Rvs: 5-CTCGAGTTTCTTGGGTTGTAAG-3′ human CCL2:

Fwd, 5′-CATAGCAGCCACCTTCATTC-3′ Rvs: 5′-GGTCAGCACAGATCTCCTTG-3′ human TNFα:

Fwd, 5′-TCTTCTCCTTCCTGATCGTG-3′ Rvs, 5′-GAGGGTTTGCTACAACATGG-3′ human TGFß:

Fwd, 5′-CCTGTGACAGCAGGGATAAC-3′ Rvs, 5′-GGAGCTGAAGCAATAGTTGG-3′ human IL-10:

Fwd, 5′-TACAGCTCAGCACTGCTCTGT-3′ Rvs, 5′-AGTTCACATGCGCCTTGATG -3′

Gene expression was normalized with GAPDH mRNA content and differences were calculated with the delta-delta (ΔΔ) Ct method, as in previous studies [12], according to the following formula: (ΔΔCt = [(Ct G.O.I.Ctl - Ct HK.G.Ctl) - (Ct G.O.I.STIM. -Ct HK.G.STIM.)]. Comparison of the expression of each gene between its control and stimulated/siRNA transfected states was determined by ΔΔCt. Results were then transformed into fold variation measurements: fold increase -2ΔΔCt. For IL-8, RPL13a was also used, in parallel to GAPDH, as a housekeeping gene.

### Enzyme-linked immunoassay (ELISA)

Mo-DCs were collected on day 7, counted and incubated at 10^6^ cells/ml in RPMI+ 0.2% BSA. Cells were incubated for 20 min with inhibitors when mentioned (5 μM SB 216763, 10 μM PTP1B inhibitor, 100 nM PP2) or their vehicle (DMSO) and stimulated for 10 h, at 37 °C, 5% CO_2_. Cells were agitated every 30–45 min to avoid pellet formation. After stimulation, cells were centrifuged and supernatants were kept, aliquoted, at − 80 °C until subsequent utilization. ELISA was performed according to manufacturer’s protocols.

### Luciferase assays

HEK-PAFR were plated 12 h before transfection in 24-well plates. Cells were transiently transfected with 200 ng of pcDNA3-hPTP1B and 40 ng of luciferase reporter constructs (see plasmid section) per well using 0.75 μl of TransIT LT1 transfection reagent according to the manufacturer’s instructions. After 8 h in DMEM 5% FBS without puromycin, medium was changed and cells were incubated in DMEM 0.2% BSA for 16 h. When mentioned, cells were incubated for 20 min with inhibitors (10 μM PTP1B inhibitor, 5 μM SB 216763) or their vehicle (DMSO). The cells were lysed 6 h after stimulation with 100 nM PAF. Luciferase activity in lysates was measured as described before [64] using a Sirius luminometer (Berthold detector systems, Montreal, QC, Canada).

### Plasmids

The human IL-8 luciferase constructs, pGL3-IL-8wt, pGL3-IL-8-ΔAP-1 (deleted for the AP-1-binding site) or GL3-IL-8-ΔC/EBPß (deleted for AP-1-binding C/EBPß) were kindly provided by Dr. Allan R. Brasier from University of Texas Medical Branch [65]. The p-NF-κB-luc reporter plasmid was kindly provided by Dr. Patrick McDonald (Université de Sherbrooke) [66]. The WT PTP1B construction and its dominant negative form (D181A) are described elsewhere [63].

### Western blot analysis

After stimulation, the reaction was stopped on ice with ice-cold PBS, cells were collected, centrifuged and lysed by adding ice-cold lysis buffer containing inhibitors (50 mM Tris HCl, pH 7.4, 150 mM NaCl, 1 mM EDTA, 1%Triton X-100, 2 mM NaF, 4 mM Na_3_VO_4_, 10 μg/ml leupeptin, 2 μg/ml pepstatin and cOmplete, Mini Protease Inhibitor Tablet) for 20 min at 4 °C under agitation, before being frozen at − 80 °C.When the samples were thawed, they were centrifuged, concentrations were determined with the Coomassie Protein Assay reagent (Thermo Scientific), according to the manufacturer’s instructions, and 4x loading buffer (40% glycerol, 258 mM Tris-HCl pH 6.8, 8% SDS, 0.008% bromophenol blue, 20% 2-mercaptoethanol) was added to the supernatants. Then, proteins were separated on 10% SDS-PAGE and subsequently transferred to a nitrocellulose membrane (Hybond, GE). Membranes were blocked with 5% milk in Tris-buffered saline (TBS) with 0.05% Tween 20 (TBS-T) before overnight blotting at 4 °C in TBS-T 5% milk with appropriate antibodies. For phospho-proteins, membranes were washed 3x5min in TBS-T after blocking in milk and blocked again for 20 min in TBS-T 5% BSA before overnight blotting at 4 °C in BSA. Finally, HRP-coupled secondary antibody (Cell Signaling) was added for 1 h, protein expression was revealed by ECL chemiluminescence detected with Versadoc (BioRad). Signal intensity was analyzed with ImageJ 1.43X software (National Institutes of Health, Bethesda, MD, USA). Data obtained were normalized and shown in fold increases over unstimulated as calculated using:

Fold Increase:

$$ \frac{Normalizedphospho- pr\mathrm{o} tein{levels}_{stimulatedcells}}{Normalizedphospho- pr otein{levels}_{unstimulatedcells}} $$

where normalized phospho-protein levels were calculated as

$$ \frac{phospho- proteinlevels}{total\mathrm{l} evelsoftheprotein} $$

## Results

### PTP1B modulation of PAF-induced cytokine expression

We had shown that PAF induces the activation of PTP1B via a pathway involving Jak2 and calpain [63]. In this report, we investigated the role of this phosphatase in the signaling pathways of PAF-induced cytokine production, focusing on IL-8 in immature monocyte-derived DCs (iMo-DCs). First, we examined whether PTP1B modulated mRNA expression of several key cytokines involved in atherosclerosis. As PAF had been shown to modulate, in several cell types, either directly or as a priming agent, the production of TNFα, CCL2, IL-10 and TGFß, all known for their atherogenic or anti-atherogenic effects, we examined the impact of the modulation of PTP1B activity, on their expression, in PAF-stimulated cells.

**Fig. 1:**
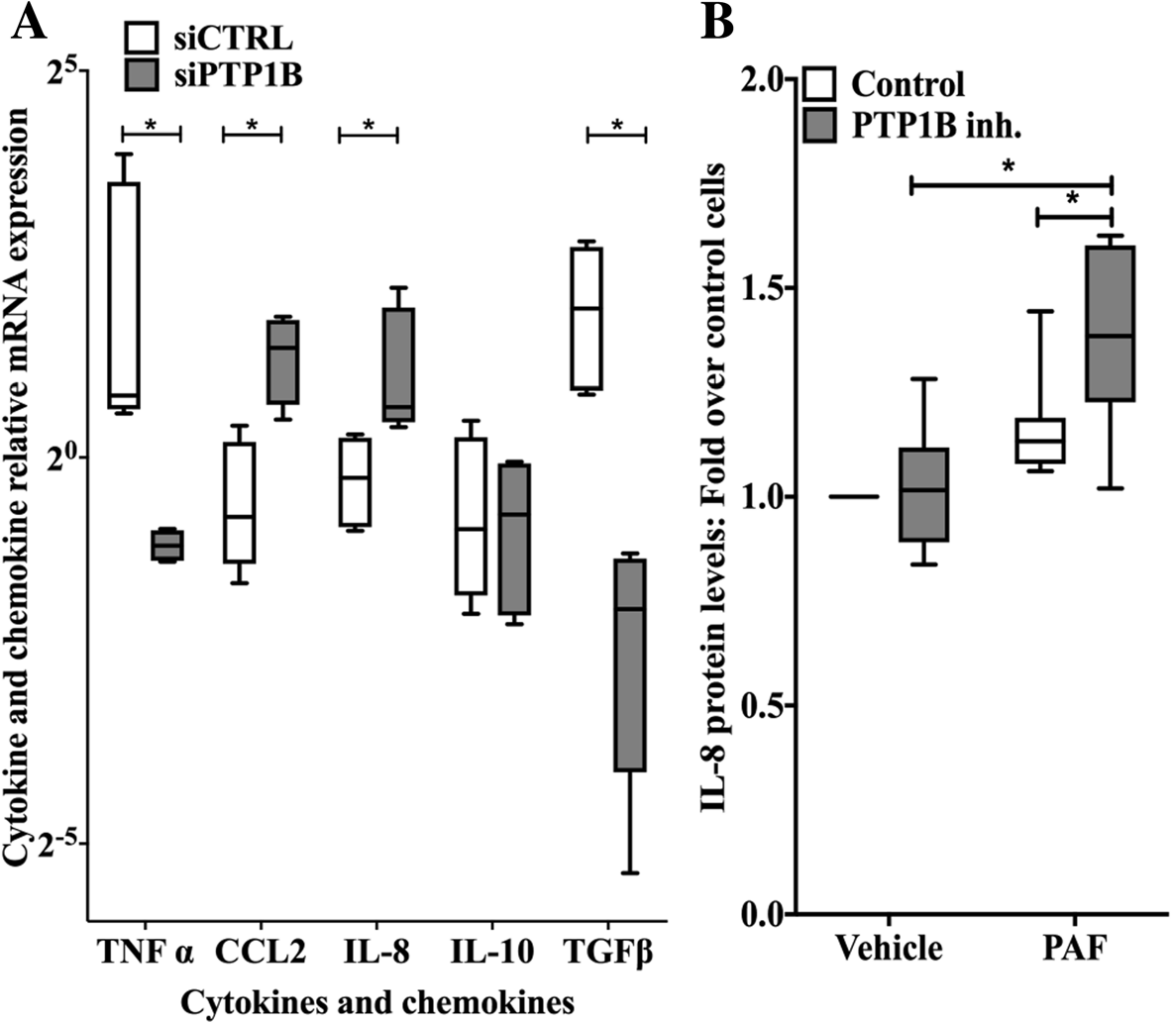
PTP1B modulation of PAF-induced cytokine expression. **a** iMo-DCs were transfected with control (siCTRL-MoDCs) or PTP1B-specific (siPTP1B-MoDCs) siRNAs on day 4 and 5. Cells were collected on day 7 and stimulated with 10 nM PAF for 5 h. Cells were lysed in Trizol and RNA was extracted and converted to cDNA. GAPDH, TNFα, IL-6, IL-8, CCL2, TGFß, IL-10 mRNA were quantified by Real-time PCR. Data are presented as mean ± S.E.M of mRNA expression calculated by the delta-delta (ΔΔ) Ct method on their respective unstimulated control for 4 independent experiments. **b** On day 7, iMo-DCs were stimulated in RMPI+ 0.2% BSA with 10 nM PAF or its vehicle for 10 h, after a 20 min pre-incubation with the PTP1B inhibitor or its vehicle (DMSO). Supernatants were collected and IL-8 levels were measured by sandwich ELISA. Data are presented in Box-and-whisker (min to max) plot graph of ratios of IL-8 concentrations in supernatants over concentrations in unstimulated, control cells (IL-8 concentration in unstimulated cells: 6.70 ± 2.46 ng/ml (mean ± S.D) and 6.92 ± 2.68 ng/ml for 10^6^ cells per ml, for vehicle and PTP1B inh. Pre-treated cells respectively). **a & b** Significance was established with paired two-way ANOVA with Sidak post-test: *:*p* < 0.05. *N* = 7

In order to study the impact of PTP1B, we used siRNAs against PTP1B or a PTP1B inhibitor (PTP1B inhibitor, Calbiochem) in PAF-stimulated iMo-DCs. We had established, previously, that the activation and maturation of the cells were not changed by the siRNA transfection and that the use of the PTP1B inhibitor gave the same results as the specific siRNAs [63]. In addition, we had demonstrated that siCTRL did not affect the expression of PTP1B compared to untransfected iMo-DCs, whereas siPTP1B down-regulated PTP1B mRNA and protein expression without affecting the expression of the highly related TC-PTP [63] or cell survival (Additional file 1: Figure S1A). Using real-time PCR, we examined cytokine mRNA expression from PAF-stimulated iMo-DCs transfected with siCTRL (siCTRL-MoDCs) or siPTP1B (siPTP1B-MoDCs). PTP1B inhibition did not modulate mRNA basal levels (Additional file 1: Figure S1B) and, to our surprise, did not modulate the mRNA expression of all cytokines examined, in the same manner (Fig. 1a). TNFα and TGFß mRNAs were decreased by PTP1B down-regulation, which resulted in a significantly lower levels of TNFα found in supernatants of iMo-DCs stimulated with PAF, but not with LPS (Additional file 1: Figure S1C). On the other hand, CCL2 and IL-8 mRNAs were increased whereas IL-10 mRNA was not modulated (Fig. 1a). These results suggested that, in iMo-DCs, PTP1B could differentially regulate cytokine expression in response to PAF stimulation. In this report, we decided to focus on PTP1B-mediated cytokine attenuation as seen with IL-8, given its importance in atherosclerosis, leaving the study of PTP1B-mediated cytokine increase (TNFα and TGFß) for a subsequent study.

In order to examine protein expression, iMo-DCs were pre-treated with the PTP1B inhibitor (PTP1B inh.), an inhibitor which is highly selective for PTP1B over TC-PTP (IC_50_ 18 μM vs > 100 μM, respectively) [67], or its vehicle (Control) for 20 min before a 10 h stimulation with PAF. As expected from results obtained with mRNA expression, the IL-8 levels found in supernatants were higher in 10 nM PAF-stimulated iMo-DCs pretreated with the PTP1B inhibitor than in stimulated control cells (Fig. 1b). A similar modulation of PAF-induced IL-8 expression was obtained when intracellular IL-8 levels were measured, suggesting that observed effects on IL-8 supernatant levels were not due to a secretory defect (Additiona file 1: Figure S1C). Moreover, the PTP1B inhibitor modulated neither lipopolysaccharide (LPS)-induced IL-8 intracellular (Additiona file 1: Figure S1D) nor TNFα secretion levels (Additional file 1: Figure S1C), suggesting a selective effect only on specific PAF-induced signaling pathways.

### PTP1B attenuation of PAF-induced IL-8 activation depends on the C/EBP binding site

**Fig. 2:**
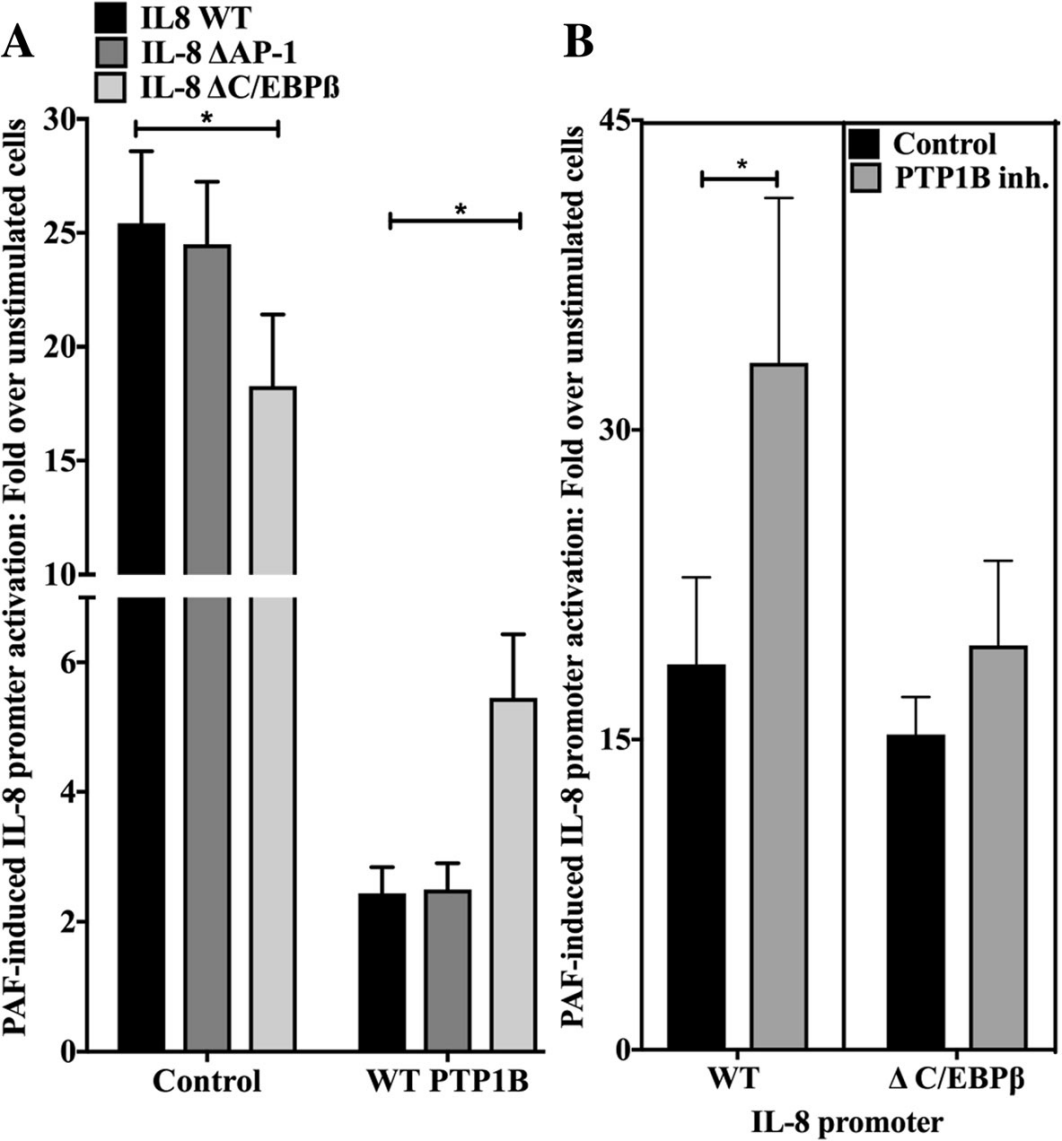
PTP1B attenuation of PAF-induced IL-8 promoter activation depends on a C/EBPß binding site. **a** HEK-PAFR were co-transfected with WT PTP1B or vector control and pGL3-IL-8 WT or deleted for C/EBPß-binding site (ΔC/EBPß) or AP-1-binding site (ΔAP-1). Cells were starved overnight in DMEM+ 0.2% BSA and stimulated for 6 h with PAF (100 nM) or vehicle and luciferase activity was measured. Data are presented as mean ± S.E.M of fold increase of at least 3 experiments. Significance was established with paired two-way ANOVA with Sidak post-test: *:p < 0.05. **b** HEK-PAFR were transfected with pGL3 IL-8 WT or ΔC/EBPß, incubated for 20 min with 10 μM PTP1B inhibitor or its vehicle, then stimulated for 6 h with PAF (100 nM) and luciferase activity was measured. Results are presented as the % of variation of PAF-induced fold increase of promoter activity caused by PTP1B inhibitor. Stimulated control cell promoter activity was set at 1. Data are presented as mean ± S.E.M of at least 4 experiments. Significance was established for pGL3-IL-8 WT and ΔC/EBPß with Mann-Whitney test. *:*p* > 0.05

We first examined if PTP1B could affect PAF-induced IL-8 transcription. We transfected HEK-PAFR, (HEK-293 cells stably transfected with the PAF receptor) with the human IL-8 promoter coupled to a luciferase gene. We studied whether or not increased PTP1B expression affected PAF-induced promoter activation. As seen in Fig. 2a, PAF-induced WT IL-8 promoter activation was significantly decreased by the co-expression of PTP1B cDNA (*p* > 0.01). We ensured that this decrease was not caused by variations of basal promoter transcription levels, due to PTP1B over-expression. As shown in Additional file 1: Figure S2A, PTP1B did not significantly modulate basal activity of the IL-8 promoter in unstimulated cells.

Next, since the IL-8 promoter is known to be under control of the NFκB transcription factor [68], the involvement of PTP1B in PAF-induced activation of NFκB was assessed. We had already shown that PAF-induced IL-8 promoter activation is abrogated when the NFκB binding site is deleted [69], thus the involvement of PTP1B in this pathway was investigated using different tools. As shown in Additional file 1: Figure S2B) and C) , PTP1B had an effect on basal activity of NFκB, as indicated by the increase in basal activation of the NFκB reporter plasmid which correlated with a decrease in IκBα levels. In stimulated cells, the IκBα degradation was comparable in controls and PTP1B-transfected cells, while the activation of the NFκB reporter was significantly increased in PTP1B-transfected cells. Altogether, these results suggest that PTP1B would not negatively regulate PAF-induced IL-8 promoter activity via the NFκB pathway.

We then used IL-8 mutant promoters where the binding site for AP-1 or C/EBPß was deleted (ΔAP-1 or ΔC/EBP) to pursue our investigation. The deletion of the AP-1 binding site in the IL-8 promoter had no significant effect on PAF-induced promoter activation or on PTP1B-mediated effects (Fig. 2a). The deletion of the C/EBPß binding site slightly, but significantly, decreased PAF-induced IL-8 promoter activation in control cells (− 25.8 ± 16.2%, mean ± S.D., *p* < 0.05). On the other hand, the deletion of this binding site reduced the downregulation of IL-8 promoter due to PTP1B over-expression; the remaninin PTP1B effects were mainly due to a slight, but non-significant, increase in basal IL-8 promoter activity caused by PTP1B over-expression (Additional file 1: Figure S2A). We concluded that the inhibitory effect was reduced, when the C/EBP binding site was absent. To avoid artefacts due to PTP1B over-expression, experiments were repeated with cells pre-treated with the PTP1B inhibitor (Fig. 2b). The pre-treatment caused a significant increase of 56.3 ± 33.06% (mean ± SD) in PAF-induced IL-8 WT promoter activity, however, the PTP1B inhibitor had little effect on IL-8 ΔC/EBPß promoter activation (14.9 ± 0.3%). These data suggest that PTP1B is an important down-regulator of PAF-induced IL-8 promoter activation and that its maximal effect depends on the presence of the C/EBPß binding site in the promoter.

### PTP1B inhibition increases PAF-induced pThr235 C/EBP levels via GSK-3 in HEK-PAFR

**Fig. 3:**
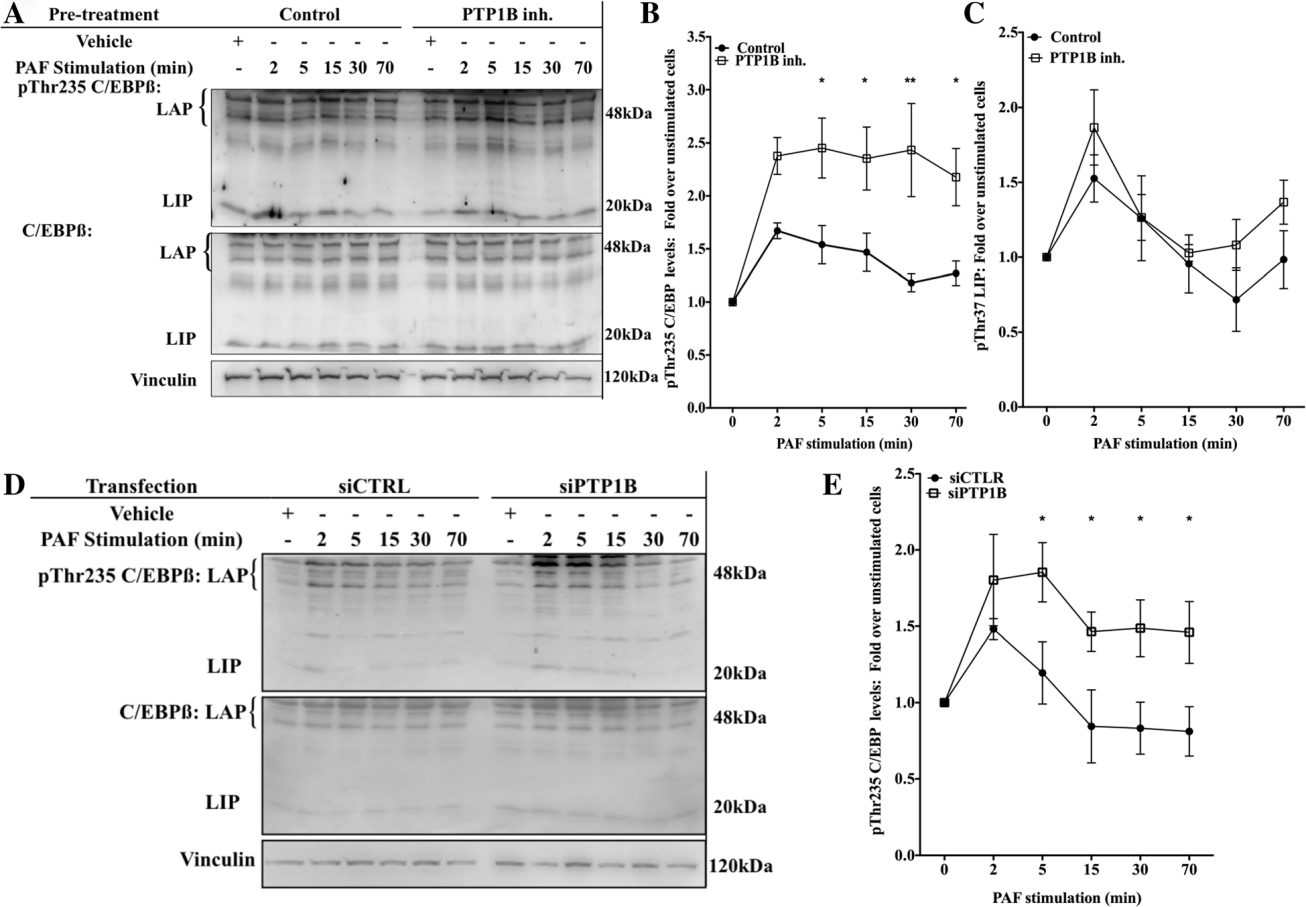
PTP1B decreased PAF-induced C/EBPß phosphorylation levels. **a-c** iMo-DCs, collected on day 7, were incubated at 10^6^ cells/ml in RPMI+ 0.2% BSA for 5 h. Cells were then incubated for 20 min with 10 μM PTP1B inhibitor before being stimulated with 10 nM PAF for indicated times. **d & e** siCTRL-MoDCs or siPTP1B-MoDCs were collected on day 7 and incubated at 10^6^ cells/ml in RPMI+ 0.2% BSA for 5 h. Cells were then stimulated for indicated times with 10 nM PAF. **a-e** Reaction was stopped on ice and cells were collected and lysed. Lysates were separated on SDS-PAGE gels and Western blots were performed with anti-vinculin, anti-pThr235 C/EBPß (LAP1 and LAP2), which also recognizes pThr37 LIP, and anti-C/EBPß antibodies (Abs). **a** A replicate blot is shown with anti-C/EBPβ antibodies (Abs) and anti-vinculin. **d** The blots were stripped between re-blotting with indicated antibodies. Representative blots are shown for each condition and compilations of at least 3 independent experiments are presented as mean ± S.E.M of normalized ratios pTh235 C/EBPß levels calculated as described in Methods. Significance was established with paired two-way ANOVA with Sidak post-test: *p > 0.05, **p > 0,01 over control cells

We therefore focused on the C/EBPß pathway. First, we examined whether the phosphorylation levels of Thr235 of C/EBPß were modulated by PTP1B, in a PAF-dependent context, given that the phosphorylation of this site had been shown to have a role in PGE2-mediated IL-8 modulation in astrocytoma [70]. iMo-DCs were pre-treated with the PTP1B inhibitor and then stimulated with PAF for indicated times. Western blots were performed using anti-pThr235 C/EBPß Abs which recognize both LAP isoforms of C/EBPß when phosphorylated at Thr235: C/EBPß-1 or LAP-1 (52 kDa in human) and LAP-2, which appears in Western blot experiments as a doublet at 48 and 45 kDa due to post-translational modifications [47]. Given the various post- translational modifications possible that can modify LAP molecular weights [47], we combined the variations of phosphorylation levels for both LAP isoforms. Results showed that PAF induced a moderate but rapid increase in the phosphorylation of Thr235 levels in control iMo-DCs (Fig. 3a and b). The inhibition of PTP1B significantly increased these phosphorylation levels from 5 to 70 min post-stimulation.

Moreover, antibodies used also recognize the LIP isoform (20 kDa), which lacks the trans-activation domain. According to their specifications, these antibodies recognize LIP only when it is phosphorylated at Thr37. Results obtained showed that this C/EBPß isoform was expressed in iMo-DCs (Fig. 3a) and PAF increased the Thr37 phosphorylation levels. As this phosphorylation site is found in a conserved MAPK consensus site, shared with the LAP isoforms [71], we also monitored the effect of PTP1B inhibition on the kinetics of its phosphorylation. The compilation of data from 4 different donors showed that PAF induced a rapid and transitory phosphorylation of LIP on Thr37 but this phosphorylation was not modulated by PTP1B inhibition (Fig. 3c). Hence, we focused on the LAP isoforms for subsequent experiments. Results obtained with the PTP1B inhibitor were confirmed with siRNA against PTP1B (Fig. 3d and e). As expected, PAF increased the levels of pThr235 detected in siCTRL-MoDCs in a time-dependent manner and this increase was significantly higher after 5 min of stimulation in siPTP1B-MoDCs than in siCTRL-MoDCs, stimulated for the same period.

Since HEK-PAFR and iMo-DCs differ in many ways, we confirmed that the increased PAF-induced IL-8 promoter activation due to PTP1B inhibition could be due to an increased phosphorylation levels of Thr235 of C/EBPßs in HEK-PAFR, as found in iMo-DCs. In HEK-PAFR, PAF increased the phosphorylation of Thr235 LAP, modestly, but significantly, as early as 2 min post-stimulation (Additional file 1: Figure S3A and B) and the inhibition of PTP1B significantly increased it at 5, 10 and 15 min post-stimulation. Together with the results obtained in luciferase assays using the C/EBPß site deletion mutant, these results indicate that PTP1B can decrease PAF-induced LAP activation, hence attenuating IL-8 promoter activation.

It has been shown that the phosphorylation of Thr235 of C/EBPß can be modulated by the Ras/ERK, p38MAPK and GSK-3 pathways [54, 70, 72, 73]. We therefore investigated which of these pathways could be regulated by PTP1B in PAF-stimulated cells. Since PAF can activate ERK in a Ras/Raf-1-dependent manner [74–76], and given that PTP1B can modulate Raf-1, both in vitro and in vivo, leading to the modulation of ERK activity [77, 78], we first investigated ERK activation. We examined pERK in PAF-stimulated iMo-DCs (Additional file 1: Figure S4A), however, this phosphorylation was not modulated by PTP1B inhibition. This was confirmed in PAF-stimulated HEK-PAFR with PTP1B inhibitor (Additional file 1: Figure S3C and D). These results suggest that pathways leading to ERK activation are not targeted by PTP1B in PAF-stimulated cells and are thus not involved in the modulation of Thr235 of C/EBPß phosphorylation levels by PTP1B.

PAF can also up-regulate p38MAPK activation [61, 79–81] and PTP1B has been shown to modulate p38MAPK activity, directly or indirectly, in other stimulatory contexts [82]. Consequently, we examined the kinetics of p38MAPK activation. An increase in phosphorylation was detected as early as 2 min after PAF stimulation (Additional file 1: Figure S4B). The inhibition of PTP1B significantly reduced the activation of p38MAPK. In addition, complementary results obtained with HEK-PAFR treated with the PTP1B inhibitor showed a reduced p38MAPK phosphorylation (Additional file 1: Figure S3E and F). Altogether, modulation of PTP1B activity by the PTP1B inhibitor or by over-expressed WT PTP1B, did not regulate p38MAPK or ERK phosphorylation in a manner consistent with PTP1B effects on PAF-induced C/EBPß phosphorylation, thus excluding the possibility of a direct phosphorylation of C/EBPß by these two MAPKs in this PTP1B-dependent pathway.

**Fig. 4:**
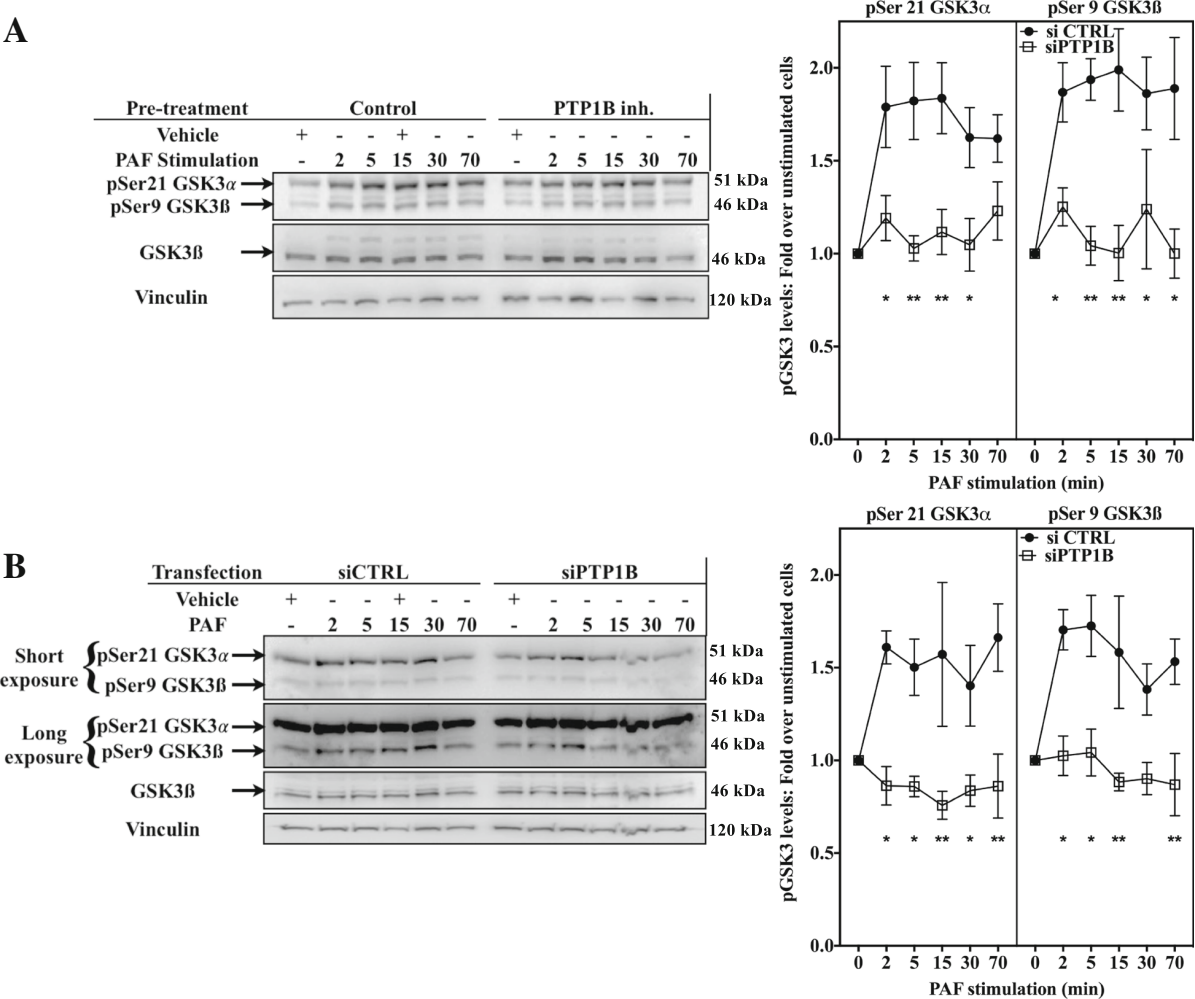
PTP1B increases PAF-induced GSK-3 phosphorylation. **a** iMo-DCs collected on day 7 were incubated at 10^6^ cells/ml in RPMI+ 0.2% BSA for 5 h, then incubated for 20 min with 10 μM PTP1B inhibitor before being stimulated with 10 nM PAF for indicated times. Reaction was stopped on ice and cells were collected and lysed. **b** siCTRL-MoDCs or siPTP1B-MoDCs were collected on day 7, incubated at 10^6^ cells/ml in RPMI+ 0.2% BSA for 5 h and stimulated for indicated times with 10 nM PAF before being lysed. **a** and **b** Lysates were separated on SDS-PAGE gels and Western blots were performed with anti-vinculin, anti-pSer21/Ser9 GSK-3 and anti-GSK-3ß Abs. The blots were stripped between re-blotting with indicated antibodies. Representative blots are shown for each experimental condition and compilations of at least 3 independent experiments are presented as mean ± S.E.M of normalized ratios of pSer21/9 GSK-3 calculated as described in Methods. Significance was established with paired two-way ANOVA with Sidak post-test: *p < 0.05, **p < 0.01 vs control cells

Next, we examined whether PTP1B could modulate the GSK-3 pathway, since PAF, alone or in combination with other stimuli, has previously been shown to modify its activity [83–87]. Furthermore, PTP1B has also been shown to regulate this pathway [88, 89]. Given that GSK-3 is a kinase with a high basal activity, many stimuli, such as PAF and growth factors, modify its activity via the modulation of the phosphorylation of the residues Ser21 for GSK-3α and Ser9 for GSK-3ß. This phosphorylation results in a decreased capacity of GSK-3 to bind primed substrates and restrains its activity [83, 90]. We first examined the phosphorylation levels of GSK-3 on Ser21/Ser9 in iMo-DCs stimulated with PAF after pre-incubation with the PTP1B inhibitor or its vehicle (control). In control cells, PAF induced a significant increase in pSer21/Ser9 GSK-3 levels as early as 2 min post-stimulation for both isoforms of GSK-3 (Fig. 4a). This increase was maintained for all tested times, suggesting that PAF induced a prolonged decrease in GSK-3 activity. Inhibition of PTP1B with the PTP1B inhibitor (Fig. 4a), or its down-regulation by siRNA (Fig. 4b), significantly decreased PAF-induced phosphorylation levels of both isoforms, suggesting that PTP1B could be involved in PAF-mediated increase of Ser21/Ser9 phosphorylation levels in iMo-DCs. Results obtained with HEK-PAFR, corroborated these results with the PTP1B inhibitor reducing GSK-3 phosphorylation of both isoforms (Additional file 1: Figure S3G-I). These results suggest that PTP1B may be involved in the negative regulation of these kinases.

**Fig. 5:**
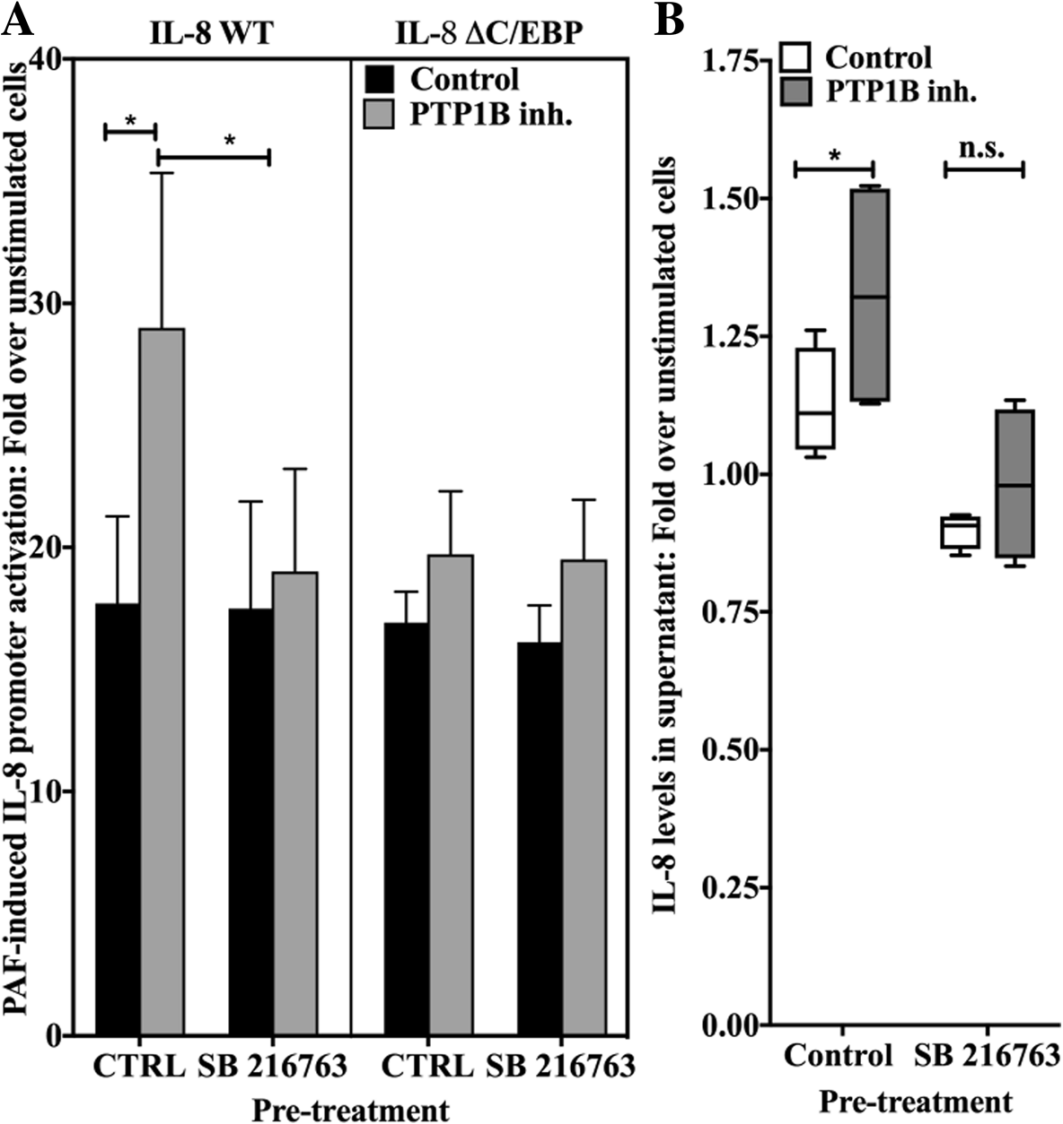
PTP1B attenuates PAF-induced IL-8 promoter activation in a GSK-3- and C/EBPß-dependent manner. **a** HEK-PAFR were transfected with pGL3-IL-8WT or ΔC/EBPß promoter reporter plasmids, incubated 20 min with 10 μM PTP1B inhibitor or 5 μM SB 216763, alone or combined with the PTP1B inhibitor, or their vehicles, then stimulated for 6 h with PAF (100 nM) and luciferase activity was measured. Results are presented as the variation of the PAF-induced fold increase of promoter activity caused by PTP1B inhibitor, SB 216763 or their combination compared to the PAF-induced IL-8 promoter activation in control cells, set to 1. Data are presented as mean ± S.E.M of at least 4 experiments. **b** iMo-DCs were collected on day 7, pre- incubated at 10^6^ cells/ml for 20 min with 10 μM PTP1B inhibitor, 5 μM SB 216763, alone or in combination with the PTP1B inhibitor, or with their vehicles. Cells were stimulated for 10 h with 10 nM PAF before supernatants were collected. IL-8 levels were measured by sandwich ELISA. Data are presented as mean ± S.E.M of ratios of IL-8 in supernatants over unstimulated control cells, for 4 independent experiments. IL-8 levels in supernatant were: 5.28 ± 1.82 ng/ml (mean ± S.D.), 5.90 ± 3.56 ng/ml, 5.81 ± 2.68 ng/ml and 5.71 ± 3.70 ng/ml for 10^6^ cells per ml pre-treated with vehicles, PTP1B inhibitors, SB216763 and PTP1B inhibitor & SB 216763, respectively **a & b** Significance was established with paired two-way ANOVA with Sidak post-test: *p < 0.05, **p < 0.01

To underscore the relevance of GSK-3 phosphorylation levels to the modulation of PAF-induced IL-8 promoter activation, we examined whether the inhibition of PAF-induced IL-8 promoter activation by PTP1B was C/EBPß- and GSK-3-dependent. We performed IL-8 promoter luciferase assays in HEK-PAFR pre-treated with either vehicle, the PTP1B inhibitor, the GSK-3 inhibitor SB 216763 or both. Experiments were done with the wild-type or mutant promoters (ΔC/EBP). Results in Fig. 5a) show the changes induced by the different inhibitors in PAF-stimulated cells. The PTP1B inhibitor induced a significant increase in PAF-induced IL-8 WT promoter activity and this increase was abrogated by the addition of the GSK-3 inhibitor. The inhibitor alone, did not significantly affect the PAF-induced promoter activation, which is consistent with the results obtained by Western blots showing that PAF tended to decrease the activation of GSK-3. When cells were transfected with the IL-8 ΔC/EBP promoter, the PTP1B inhibitor alone, as was shown in Fig. 2b), did not increase the PAF-induced promoter activity and the addition of the GSK-3 inhibitor, also, did not have any significant effect, Fig. 5a). These results suggest that the negative modulation of PTP1B on PAF-induced IL-8 promoter activity depends on GSK-3 activity and the presence of an intact binding-site for C/EBPß.

Next, the importance of GSK-3 in the modulation of PTP1B activity in PAF-induced IL-8 expression was assessed in iMo-DCs. The cells were incubated with either the vehicles, the PTP1B inhibitor, the GSK-3 inhibitor SB216763 or both inhibitors combined, before stimulation with PAF. Supernatants were then collected and IL-8 protein was quantified by ELISA, Fig. 5b shows that PAF, as expected, increased IL-8 protein levels with a significant augmentation in the presence of the PTP1B inhibitor. SB216763 slightly, but significantly decreased PAF-induced IL-8 levels and the PTP1B inhibitor did not overcome the SB216763-mediated inhibition. Taken together, these results suggest that the increase in IL-8 levels brought about by PTP1B inhibition in iMo-DCs depends on GSK-3 activity.

**Fig. 6:**
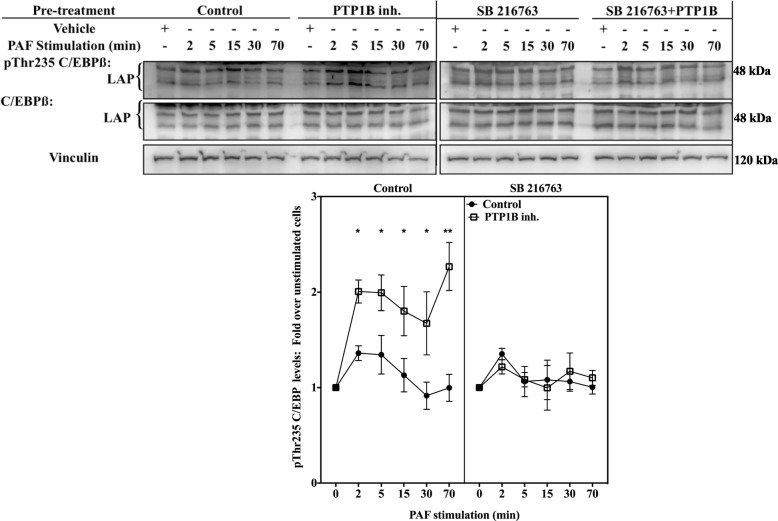
PTP1B decreases PAF-induced C/EBPß phosphorylation in a GSK-3-dependent manner. iMo-DCs were collected on day 7, incubated for 20 min with 10 μM PTP1B inhibitor, 5 μM SB 216763, alone or in combination, or with their vehicles. Cells were then stimulated with 10 nM PAF for indicated times. Reaction was stopped on ice and cells were collected and lysed. Lysates were separated on SDS-PAGE gels and Western blots were performed using Abs recognizing vinculin, pThr235 C/EBPß and C/EBPß. Control and SB216763-treated cells are shown on two different blots. The blots were stripped between re-blotting with indicated antibodies. Control experiment samples are the same as in Fig. 3a. Representative blots are shown for each experimental condition and compilations are presented as mean ± S.E.M indices of normalized ratios of pThr235 C/EBPß levels calculated as described in Methods, of at least 3 experiments. Significance was established with paired two-way ANOVA with Sidak post-test: *p < 0.05, PTP1B **p < 0,01 vs control cells

We then examined whether the decreased GSK-3 phosphorylation, following PTP1B inhibition, resulted in a change of C/EBPß phosphorylation (Thr235). Lysates were obtained from iMo-DCs, after pre-incubation with vehicles, the PTP1B inhibitor or SB216763 alone or in combination, followed by stimulation with PAF, for indicated times. The phosphorylation levels of C/EBPß-Thr235 were significantly increased after PAF stimulation in the presence of the PTP1B inhibitor, and inhibition of GSK-3 by SB216763 impaired this increase (Fig. 6), suggesting that the increase of pThr235 levels, consequent to PTP1B inhibition, may depend on GSK-3 activity.

### PTP1B decreases pThr235 C/EBPß levels in Mo-DCs via a SFK pathway

**Fig. 7:**
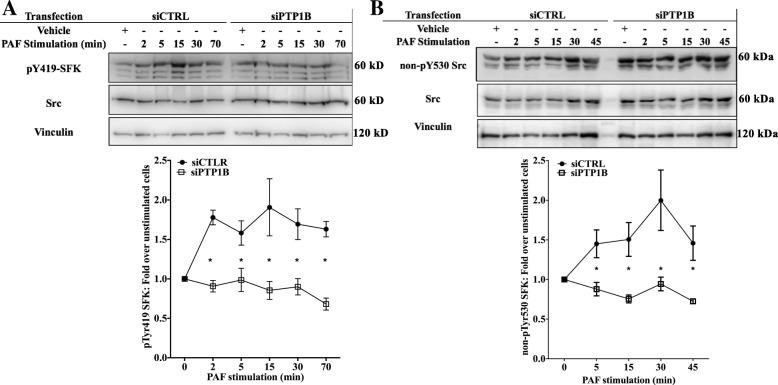
PTP1B increases PAF-stimulated SFK activation in iMo-DCs. **a** and **b** siCTRL-iMoDCs or siPTP1B-iMoDCs were collected on day 7 and stimulated for indicated times with 10 nM PAF. Reaction was stopped on ice and cells were collected and lysed. Whole cell lysates were loaded onto SDS-PAGE, transferred to nitrocellulose membrane and blotted overnight with antibodies (Abs) recognizing vinculin, Src and **a** pTyr419 SFK or **b** non-phosphorylated form of Tyr530 of Src (non-Tyr530). The blots were stripped between re-blotting with indicated antibodies. Representative blots are shown for each experimental condition and compilations of at least 4 independent experiments are presented as mean ± S.E.M indices of normalized ratios of **a** pTyr419 SFK or **b** non-Tyr530 Src levels calculated as described in Methods. Significance was established with paired two-way ANOVA with Sidak post-test: * < 0.05, PTP1B vs control cells

Given that PTP1B regulation of serine phosphorylation levels would be indirect, we looked for a known PTP1B target molecule which could modulate a pathway leading to modified GSK-3 phosphorylation. It has been shown that PTP1B can control Src family kinases (SFK) via the de-phosphorylation of an inhibitory tyrosine (Tyr530), resulting in higher phosphorylation of the activating tyrosine (Tyr419), thus activating the kinases. The SFKs, in turn, are upstream of signaling pathways which can modulate the GSK-3-C/EBPß axis, such as Akt [91–93]. We therefore examined if PTP1B is involved in PAF-induced SFK activation. For this, iMo-DCs were transfected with siRNA directed against PTP1B or control siRNA and then stimulated with 10 nM PAF for indicated times before lysis. In siCTRL-iMo-DC, PAF induced an increase in pTyr419 SFK levels as early as 2 min post-stimulation (Fig. 7a) and plateaued thereafter. In siPTP1B-iMo-DCs, the PAF-induced pTyr419-SFK levels were significantly lower at all tested times. Results obtained with siRNA were confirmed by incubating untransfected iMo-DCs with the PTP1B inhibitor and then stimulated them with 10 nM PAF (Additional file 1: Figure S5A and B). The presence of the inhibitor resulted in a significant decrease of SFK activation, as shown by the decrease in pY419-SFK levels, confirming results obtained with the siRNA. These results suggest that PTP1B is involved in PAF-induced SFK activation in iMo-DCs. Given that PTP1B is known to activate Src by dephosphorylating the inhibiting pTyr530, we also monitored its phosphorylation levels after a PAF stimulation using siPTP1B-MoDCs and their control cells. For this, we performed Western blots, using antibodies directed against the non-phosphorylated form of Tyr530 Src. Results showed that PAF induced an increase in the non-phosphorylated form of this residue and also showed that in siPTP1BMo-DCs, this increase was eliminated (Fig. 7b), suggesting that the increase, by PTP1B, of PAF-induced SFK activation could possibly be via a direct effect of this PTP on SFKs, at least Src, via the dephosphorylation of Tyr530.

**Fig. 8:**
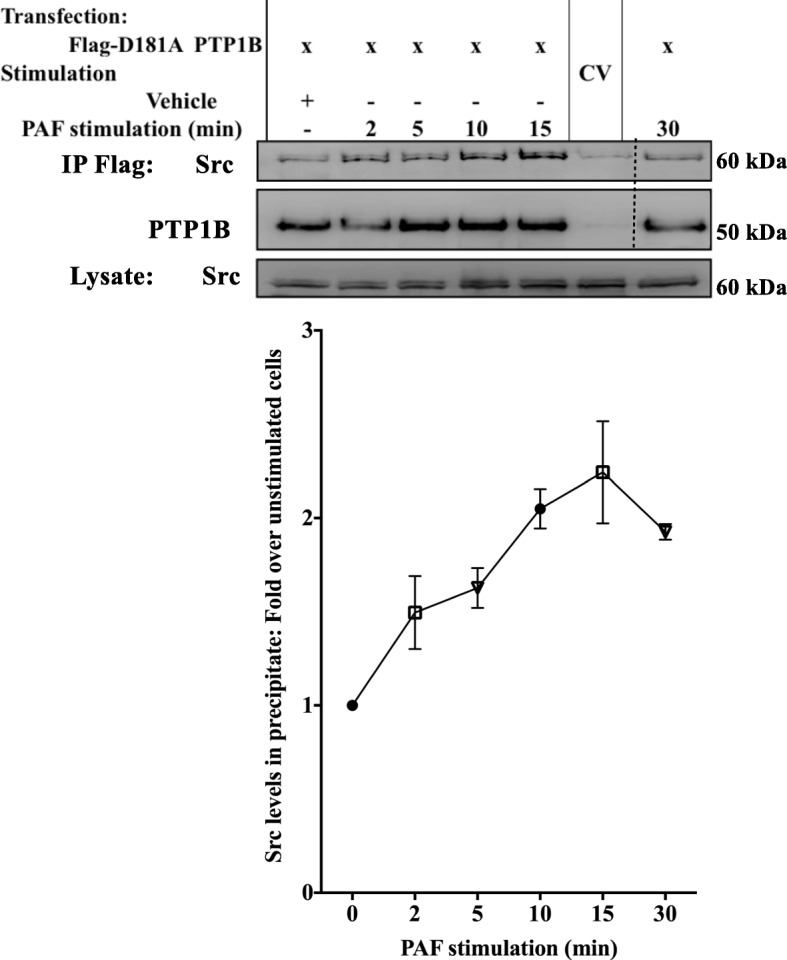
PAF increases interaction between PTP1B and Src. HEK-PAFR were transiently transfected with D181A PTP1B tagged with Flag or control vector expressing Flag alone (CV). Cells were incubated overnight in DMEM-0.2% BSA, then stimulated with PAF for indicated times. Stimulation was stopped on ice, cells were scraped and lysed. Whole cell lysates were immunoprecipitated using anti-Flag Abs coupled to agarose beads. Precipitates were loaded onto SDS-PAGE, transferred to nitrocellulose membranes and blotted overnight with anti-Src and then with anti-Flag Abs. The doted lines indicate where an empty well was removed in order to align the wells with the appropriate wells in the lysate. Representative blots and compilations of 3 independent experiments are shown. The data presented are mean ± S.E.M of normalized Src values (over PTP1B) over values for unstimulated cells

The possibility that PTP1B had a direct effect of Src was then examined by investigating if both enzymes could be found in the same signaling complex after PAF stimulation. As the interaction between phosphatases and their substrates are habitually very short, we turned to our model of HEK-PAFR transiently transfected with the D181A PTP1B cDNA after having confirmed that in this cell type, the PAF-induced SFK activity was increased by PTP1B in a similar manner to iMo-DCs (Additional file 1: Figure S5C and D). This mutant traps its substrate bound to the catalytic site, increasing the duration of the interaction between substrate and PTP. We then transfected HEK-PAFR with the control vector (C.V.) or Flag-tagged D181A PTP1B which was then immunoprecipitated after stimulation with PAF for indicated times. The amount of Src found in the precipitate of PAF-stimulated, D181A PTP1B-transfected cells increased in a time-dependent manner (Fig. 8). This indicates that PAF can induce the formation of a signaling complex where PTP1B and Src are found together and with results obtained with the decreased phosphorylation of Tyr530 Src in iMo-DCs (Fig. 7), suggests that PTP1B may directly activate SFK in PAF-stimulated cells.

**Fig. 9:**
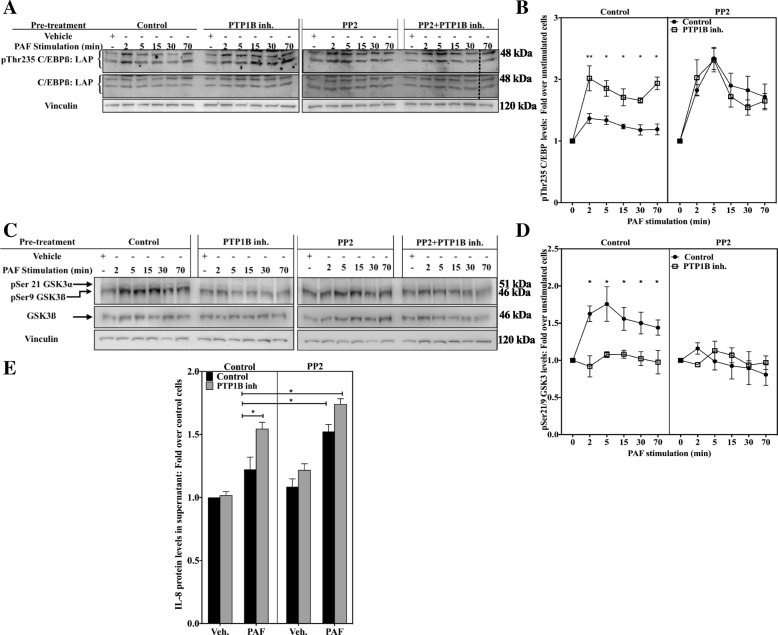
SFKs modulate PAF-induced C/EBPß and GSK-3 phosphorylation and IL-8 expression in a similar manner to PTP1B. **a-d** iMo-DCs were collected on day 7, incubated for 20 min with 10 μM PTP1B inhibitor, 100 nM PP2, alone or in combination with the PTP1B inhibitor, or with their vehicles. Cells were stimulated with 10 nM PAF for indicated times. Reaction was stopped on ice and cells were collected and lysed. Lysates were separated on SDS-PAGE gels and Western blots were performed with Abs recognizing vinculin, **a** and **b** pThr235 C/EBPß and C/EBPß or **c** and **d** anti-pSer21/9 GSK-3 or anti GSK-3ß. Representative blots are shown for each condition. Control and PP2-treated cells are shown on two separate blots and compilations of 5 independent experiments are presented as mean ± S.E.M of normalized ratios of pThr235 C/EBPß levels calculated as described in Methods. The blots were stripped between re-blotting with indicated antibodies. The doted lines indicate where the image was slightly rotated for better alignment with the rest of the blot, due to warped migration. **e** Cells were stimulated with 10 nM PAF for 10 h before supernatants were collected. IL-8 levels were measured by sandwich ELISA. Data are presented as mean ± S.E.M of ratios of IL-8 in supernatants over unstimulated control cells, for 3 independent experiments. IL-8 levels in supernatant were: 7.11 ± 1.91 ng/ml (mean ± S.D.), 7.11 ± 2.43 ng/ml, 7.53 ± 2.80 ng/ml and 8.54 ± 3.10 ng/ml for cells pre-treated with vehicles, PTP1B inhibitors, PP2 and PTP1B inhibitor & PP2, respectively **a-e** Significance was established with paired two-way ANOVA with Sidak post-test: *p < 0.05, **p < 0.01 vs control cells

We next assessed the impact of SFK inhibition on GSK-3 and C/EBPß activation levels in PAF-stimulated iMo-DCs. For this, cells were stimulated with PAF, after pre-treatment with the vehicle(s), the pan SFK inhibitor PP2, alone or in combination, with the PTP1B inhibitor. Results obtained with PP2 (Fig. 9a and b), showed a significant increase in pThr235C/EBPß, similarly to the results obtained with the PTP1B inhibitor. Furthermore, the combination of both inhibitors did not further increase the pThr235 C/EBPß levels, compared to each inhibitor alone.

Next, we assessed the impact of SFK inhibition on pSer21/Ser9 GSK-3 levels in iMo-DCs, by stimulation with PAF, after a pre-treatment with the vehicle(s), the pan SFK inhibitor PP2 alone or in combination with the PTP1B inhibitor. Results obtained with PP2 (Fig. 9c and d) showed a decrease in PAF-induced pSer21/Ser9 GSK-3 levels, similarly to what is observed with the PTP1B inhibitor alone. Furthermore, the combination of these two inhibitors did not induce any further significant modifications in the PAF-induced pSer21/Ser9 GSK-3 levels, suggesting that both inhibitors possibly decreased pSer21/Ser9 GSK-3 levels through common effectors, and this, with similar kinetics. These results, combined with those presented above suggest that PTP1B decreases GSK-3 activity leading to a decreased phosphorylation of C/EBPß on Thr235, possibly via the up-regulation of PAF-induced SFK activation.

Lastly, we examined whether the PTP1B/SFK pathway was involved in the attenuation of PAF-induced IL-8 expression. For this, iMo-DCs were incubated with either the vehicles, the PTP1B inhibitor, the SFK inhibitor PP2 or both inhibitors combined, before stimulation with PAF. Supernatants were then collected and IL-8 protein was quantified by ELISA. Figure 9e) shows that PTP1B inhibition, as expected, significantly increased PAF-induced IL-8 protein levels and SFK inhibition by PP2 recapitulated that. Furthermore, the combination of these two inhibitors did not induce any further significant modifications, suggesting that both inhibitors possibly increase IL-8 expression via the same signaling pathways. Taken together, these results suggest that PTP1B may decrease PAF-induced IL-8 expression trough the SFK/GSK-3/LAP signaling pathway.

**Fig. 10:**
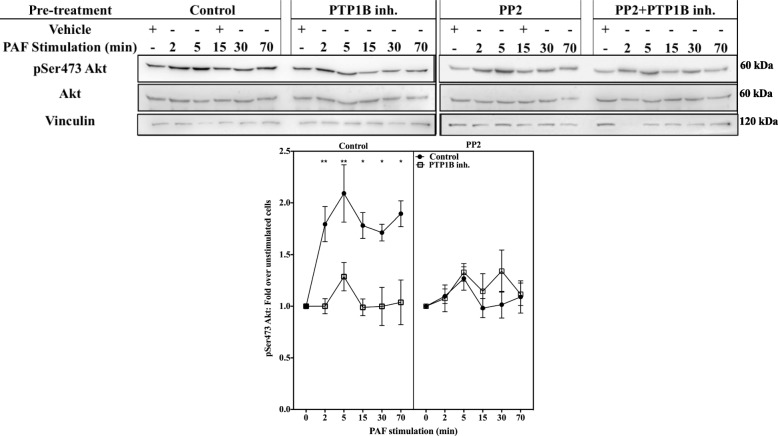
SFKs modulate PAF-induced Akt phosphorylation in a similar manner to PTP1B. iMo-DCs were collected on day 7, incubated for 20 min with 10 μM PTP1B inhibitor, 100 nM PP2, alone or in combination, or with their vehicles. Cells were stimulated with 10 nM PAF for indicated times. Reaction was stopped on ice and cells were collected and lysed. Lysates were separated on SDS-PAGE gels and Western blots were performed with anti-vinculin, anti-Ser473 Akt or anti-Akt Abs. Control and PP2-treated cells are shown on two separate blots. The blots were stripped between re-blotting with indicated antibodies. Representative blots are shown for each condition and compilation of experiments is presented as mean ± S.E.M of normalized ratios of pSer473 Akt levels calculated as described in Methods for 4 experiments. Significance was established with paired two-way ANOVA with Sidak post-test: *p < 0.05, **p < 0,01 vs control cells

To further understand the mechanisms by which the PTP1B/SFK pathway modulates GSK-3 activity, we investigated the signaling pathways upstream of the phosphorylation of GSK-3. One of the best characterized PAF-activated pathway that could lead to GSK-3 phosphorylation on Ser21/Ser9 is the PI3K/Akt pathway [90, 94, 95]. First, we explored whether PTP1B inhibition modulated the kinetics or the amplitude of Akt phosphorylation on Ser473. Results obtained with iMo-DCs pre-treated with vehicle indicated that PAF induced Akt phosphorylation as early as 2 min post-stimulation (Fig. 10 and Additional file 1: Figure S6A and B) and remained higher than basal levels until 70 min m Inhibition of PTP1B with the PTP1B inhibitor significantly reduced this phosphorylation (Fig. 10 and Additional file 1: Figure S6A and B). We confirmed the results obtained with the PTP1B inhibitor by transfecting iMo-DCs with siRNAs against PTP1B or their control siRNAs (Additional file 1: Figure S6C and D).

Next, we assessed the impact of SFK inhibition on Akt activation levels in iMo-DCs, by stimulation with PAF, after a pre-treatment with the vehicle(s), the pan SFK inhibitor PP2 alone or in combination with the PTP1B inhibitor. Results obtained with PP2 (Fig. 10) showed a decrease in PAF-induced pSer473 Akt levels, similarly to what is observed with the PTP1B inhibitor alone. Furthermore, the combination of these two inhibitors did not induce any further significant modifications in PAF-induced pSer473 Akt levels, suggesting that both inhibitors may modulate Akt activation through common effectors, and this, with similar kinetics.

**Fig. 11:**
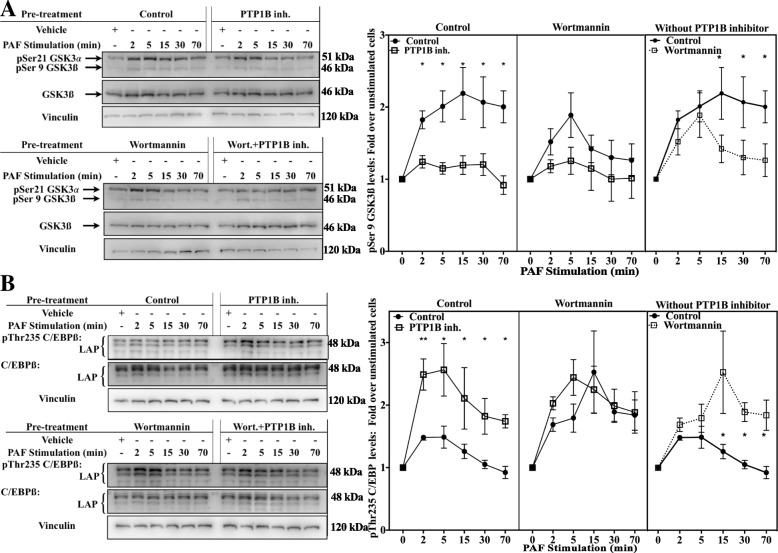
The PI3K/Akt pathway modulates GSK-3 and C/EBPß phosphorylation in a similar manner to that of PTP1B, but only at later stimulation times. **a-b** iMo-DCs were collected on day 7 and incubated for 20 min with 10 μM PTP1B inhibitor, 100 nM wortmannin, alone or in combination with the PTP1B inhibitor or with their vehicles (DMSO). Cells were then stimulated with 10 nM PAF for indicated times. Reaction was stopped on ice, cells were collected and lysed. Lysates were separated on SDS-PAGE gels and Western blots were performed with Abs recognizing vinculin and **a** pSer21/9 GSK-3 and GSK-3ß or **b** pThr235 C/EBPß and C/EBPß. The blots were stripped between re-blotting with indicated antibodies. Representative blots are shown for each experimental condition and compilations of experiments are presented as mean ± S.E.M of normalized ratios phospho-protein levels for at least 3 experiments. Significance was established with paired two-way ANOVA with Sidak post-test: *p < 0.05, **p < 0.01 vs control cells

Data presented up until now link PTP1B to both the GSK-3/C/EBPß and the SFK/Akt axis. We hypothesized that the PI3K/Akt pathway could be the missing link between these two axes and its activation could result in the modulation of GSK-3 and C/EBPß phosphorylation. To test this, iMo-DCs were incubated with vehicle(s), the PTP1B inhibitor, wortmannin (PI3K inhibitor) or both inhibitors, before stimulation with PAF. We avoided the use of LY294002, another inhibitor of PI3K, since LY294002 can affect GSK-3 activity, at least in vitro [96]. Western blot analysis (Fig. 11a) showed that the PAF-induced increase of GSK-3 phosphorylation levels was significantly lower in PTP1B inhibitor-treated cells, alone or in combination with wortmannin, for all tested times. In iMo-DCs treated with wortmannin alone, significantly lower pSer21/9 GSK-3 levels were observed only after 15 min, and this, despite a strong inhibition of Akt phosphorylation (Additional file 1: Figure S6E). These results suggest that the PI3K/Akt pathway was involved in only the late phase of GSK-3 phosphorylation and that the inhibition of PTP1B could modulate, at least at later time points, pSer21/Ser9 GSK-3 levels via the Akt pathway.

**Fig. 12:**
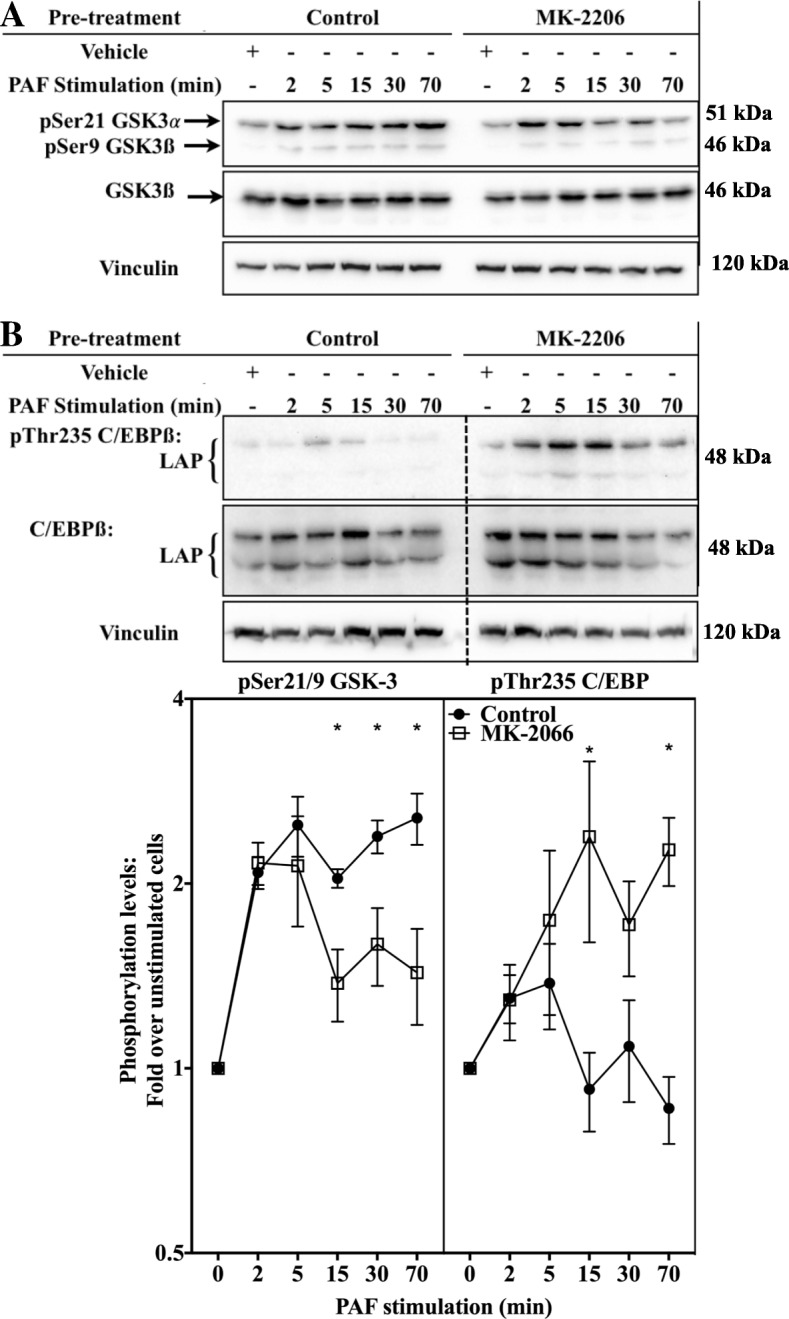
The Akt pathway modulates GSK-3 and C/EBPß but only at later stimulation times. iMo-DCs were collected on day 7 and incubated for 20 min with 2.5 μM Akt inhibitor MK-2206 or with its vehicle (DMSO). Cells were then stimulated with 10 nM PAF for indicated times. Reaction was stopped on ice, cells were collected and lysed. Lysates were separated on SDS-PAGE gels and Western blots were performed with Abs recognizing vinculin and **a** pSer21/9 GSK-3 and GSK-3ß or **b** pThr235 C/EBPß and C/EBPß. The blots were stripped between re-blotting with indicated antibodies. The doted lines indicate where the image was slightly rotated for better alignment with the rest of the blot, due to warped migration. Representative blots are shown for each experimental condition and compilations of experiments are presented as mean ± S.E.M of normalized ratios of phospho-protein levels for at least 4 experiments. Significance was established with paired two-way ANOVA with Sidak post-test: *p < 0.05 vs control cells

Consistent with the results obtained above, linking GSK-3 activity to modulations of pThr235 C/EBPß levels, incubation of iMo-DCs with wortmannin increased phosphorylation levels on this residue only at 15 min of stimulation and thereafter, (Fig. 11b) while the PTP1B inhibitor increased it at all tested times. Furthermore, the combination of both inhibitors did not change Thr235 C/EBPß phosphorylation levels compared to the PTP1B inhibitor alone, suggesting that pathways leading to the phosphorylation of this residue, in late stimulation phase that were affected by wortmannin, could already be modulated by the PTP1B inhibitor. To ensure that the late modulation observed in pSer21/9 GSK-3 and pThr235 C/EBPß levels depended on Akt and not on other PI3K effectors, we used the selective Akt inhibitor MK-2206. Consistent with results found after pre-treatment of the cells with wortmannin, Akt inhibition resulted in a significant down-regulation of the PAF-induced pSer21/9 GSK-3 (Fig. 12a and c) and a consequent up-regulation of pThr235 C/EBPß (Additional file 1, Fig. 12b and c) only at later PAF stimulation times, and this, despite a strong inhibition of Akt phosphorylation (Additional file 1: Figure S6F). Therefore, the results presented in this section suggest that during PAF stimulation, the activation of PI3K/Akt was involved in down-regulation of GSK-3 activity leading to a weaker phosphorylation of C/EBPß at later stimulation times, thus PTP1B, by positively modulating PI3K/Akt signaling, was involved in this modulation of C/EBPß phosphorylation.

Altogether, our results suggest that PTP1B is involved in PAF-induced SFK activation. This kinase family would be, in turn, implicated in the reduction of GSK-3 activity by inducing its phosphorylation via the PI3K/Akt pathway at later stimulation times and by a yet unidentified pathway at initial stimulation times. By regulating these pathways, PTP1B could control the PAF-induced phosphorylation of C/EBPß and consequently, PAF-activated IL-8 expression.

## Discussion

Given that PAF is an important bioactive molecule in the initiation and progression of atherosclerosis [28], in this report, we aimed at increasing our understanding of the signaling pathways involved in the modulation of PAF-induced cytokine expression in iMo-DCs and the potential involvement of PTP1B in its signal transduction.

We found that, in iMo-DCs, either a small but significant reduction of PTP1B expression or its inhibition by a pharmacological inhibitor was enough to perturb PAF-induced expression of CCL2, IL-8, TNFα and TGFß (Fig. 1 and Additional file 1: Figure S1). Moreover, this modulation appears to be specific to certain PAF-induced signaling pathways: indeed, PTP1B differentially modulated all the cytokines mentioned above, and this, without affecting LPS-induced IL-8 or TNFα expression, hence reducing the possibility that these modulations were due to a general loss of signal transduction or to a hyper-activation of the cells. We therefore concluded that PTP1B is important for regulation of PAF-induced cytokine expression.

We concentrated on IL-8 which contributes to the recruitment of immune cells, such as monocytes and DCs, to atherosclerotic lesions by inducing their chemotaxis and facilitating their extravasation through the retraction of endothelial cells and the expression of matrix metalloproteinases metalloproteinases [22, 23, 26, 97]. We found that PAF-induced IL-8 expression in iMo-DCs was tightly regulated in an inverse concentration-dependent manner: higher PAF concentrations induced lower IL-8 expression levels (Additional file 1: Figure S7) and PTP1B was involved in the lowering of IL-8 expression (Fig. 1). The use of the PTP1B inhibitor confirmed the importance of PTP1B phosphatase activity in the activation of the IL-8 promoter and validated results obtained by over-expression of PTP1B in HEK-PAFR. Moreover, these results were in line with siRNA-transfected or PTP1B inhibitor-treated iMo-DCs, stimulated with PAF, suggesting that PTP1B is involved in the negative regulation of IL-8 production. Thus PAF, a potent pro-inflammatory mediator, seemed to induce a negative feedback pathway when PAF concentrations were high. We focused on elucidating the signaling pathways involved in the attenuation of IL-8 production. We found that PTP1B was involved in PAF-induced SFK activation given that PTP1B inhibition, or its down-regulation, strongly impaired pTyr419 SFK levels, an activating, auto-phosphorylation site, and increased pTyr530 Src levels, a site directly dephosphorylated by PTP1B [58, 98, 99]. We also found that the SFKs could be the downstream effectors of PTP1B, given that the SFK inhibitor PP2 recapitulated the effect of PTP1B inhibition/down-regulation on the phosphorylation levels of Akt, GSK-3 and C/EBPß and on IL-8 expression. Together with co-immunoprecipitation experiments indicating that PAF induced the formation of a complex between a member of the SFK family, Src, and PTP1B, this leads us to conclude that PTP1B could directly dephosphorylate and activate SFK/Src. Since PAFR can activate EGFR [42, 81] and some GPCRs activate Src pathway in an EGFR-dependent manner [100], we excluded the possibility that PTP1B increased SFK activation indirectly through an EGFR (Epithelial-Growth Factor Receptor) -dependent pathway. We examined, in HEK-PAFR, EGFR phosphorylation levels on Tyr1068, an auto-phosphorylation site known to be a good assessment of EGFR activity and to be modulated by PTP1B [101]. We found that even if PAF significantly increased EGFR phosphorylation levels Additional file 1: Figure S8, overexpressed WT PTP1B did not modulate the phosphorylation, suggesting that PTP1B-mediated SFK activation was not due to the modulation of EGFR activity.

Data obtained here also suggest that the PTP1B/SFK pathway attenuated IL-8 production in a C/EBPß-dependent pathway given the need of the C/EBPß binding-site for the down-regulation of PAF-induced IL-8 promoter activation by PTP1B. Alone, these results should be taken with caution given that they do not rule out the possibility that the lack of significant effect of the PTP1B inhibitor on PAF-induced ΔC/EBP IL-8 promoter activation was due to the loss of the cooperation between NFκB and C/EBP for binding to their respective sites, due to C/EBP-binding site deletion [102], hence masking any increase in NFκB activity. PAF-induced IL-8 expression depends on NFκB activity in different cell types such as monocytes and HEK-PAFR [32, 69, 103, 104], but we found that PTP1B did not down-regulate NFκB activation in PAF-stimulated cells, hence excluding any direct involvement of this pathway in PTP1B-mediated IL-8 expression attenuation. Therefore, we concluded that PTP1B may attenuate IL-8 production in a C/EBPß-dependent manner in HEK-PAFR and in iMo-DCs.

Our results showed that PTP1B inhibition increased PAF-induced C/EBPß phosphorylation levels of Thr235 LAP isoforms in HEK-PAFR cells and in iMo-DCs. The phosphorylation on Thr235 was previously reported as important for the induction of sequential phosphorylation of C/EBPß, which is involved in DNA-binding activity and/or its transactivation potential: the mutation of this residue is sufficient to impair stimulation-induced DNA binding to certain promoters [54, 72] or the transactivation of others, even if the mutated form has a higher DNA binding capacity [71]. The phosphorylation of the two major isoforms of C/EBPß, LAP-1 and 2 was modulated by PTP1B at the same level. However, neither the PTP1B inhibitor (Fig. 3a and c) nor siPTP1B (Fig. 3d, *n* = 2, compilation not shown) significantly modulated the pThr37 LIP levels. Although these results must be interpreted carefully, since LIP was barely detectable in the lysates from the majority of donors, it is tempting to speculate that the lack of modulation of this truncated form could be associated with the differences in localization between the isoforms. LIP is mostly nuclear, whereas the other isoforms are also found in the cytoplasm, depending on cell types, suggesting that the phosphorylation of C/EBß by GSK-3 would be mostly cytoplasmic, in this context. This is consistent with data reporting that even if GSK-3 can be found in the nucleus, some of its effects are exclusively cytoplasmic [105–107].

It is known that the residue Thr235 of C/EBPß is the phosphorylation target of at least 3 important pathways: ERK, p38MAPK and GSK-3 [54, 70–73, 108, 109] all known to be activated by PAFR [61, 80–86, 110], whereas the Thr37 of LIP is found in a MAPK consensus site [71], which corresponds to the Thr235 of LAP isoforms. Therefore, one can hypothesize that PAF-induced ERK activation, which was independent of PTP1B activity, may result in LIP phosphorylation but also in the C/EBPß phosphorylation levels found in control iMo-DCs that still remained after inhibition of GSK3 or PTP1B. These low phosphorylation levels which were independent of PTP1B modulation, may contribute to the small increase in IL-8 expression found in cells stimulated with 10 nM PAF.

We also concluded that PTP1B did not modulate PAF-induced LAP phosphorylation via mechanisms that involved its direct phosphorylation on Thr235 by p38 MAPK because PTP1B inhibition decreased p38 activation. These results are consistent with those from other studies [111], and also suggested an indirect effect given that, both in vivo and in vitro, PTP1B can directly dephosphorylate p38MAPK, probably on Tyr182 [62, 82]. Nevertheless, the decrease of p38MAPK phosphorylation levels, after PTP1B inhibition, was not compatible with the hypothesis of direct phosphorylation of LAP by this kinase and suggested that p38MAPK would not be directly involved in PAF-induced C/EBPß phosphorylation.

Our results suggest, instead, that PTP1B and SFK formed a signaling complex that decreased GSK-3 activity, hence decreasing C/EBPß phosphorylation levels and attenuating PAF-induced IL-8 expression. GSK-3 is a complex enzyme that can be regulated by its sub-cellular localization, its binding partners, via the control of its phosphorylation levels on Tyr279/216 (GSK-3α/ß) (necessary for proper folding and catalytic activity of the protein) and via the phosphorylation on Ser21/9 [90, 91, 105, 111, 112]. Although there is some controversy, data from the literature suggest that Tyr216/219 is an intramolecular auto-phosphorylated site, inaccessible to phosphatase activity [90, 112–114]. PAF has been shown to modulate pSer21/9 GSK-3 levels in different cell types [83, 84, 87]. Therefore, we examined the modulation of pSer21/9 GSK-3 by the PTP1B/SFK pathway in PAF-stimulated cells and found, in two different cell types, using siRNAs and inhibitors, that this pathway contributed to the increase in pSer21/9 GSK-3 levels induced by PAF. Moreover, results obtained by over-expression of PTP1B (Additional file 1: Figure S9), showed that increased expression of this PTP resulted in significantly increased p38 MAPK and Ser21/9 GSK-3 phosphorylation levels and this, without affecting ERK phosphorylation levels. Altogether, these three different tools allowed us to strengthen the conclusion that PTP1B selectively modulated these PAF-induced signaling pathways.

A number of outstanding issues remain, taking into account the results presented in this report. First, these results do not allow us to exclude either the possibility that other molecules are necessary for the GSK-3-dependent phosphorylation of C/EBPß, such as APC (Adenomatous Polyposis Coli Protein) [73] or the possibility that GSK-3 does not directly phosphorylate LAP. Moreover, data presented here do not allow us to exclude that GSK-3 could sequentially phosphorylate C/EBPß on other residues, such as Ser184, to increase the transcriptional potential of C/EBPß or on other sites found in the Serine-rich region surrounding Thr235 which can also result in positive or in negative variations of the DNA-binding and transactivation potential [71, 115]. Nevertheless, using the specific GSK-3 inhibitor SB216763, we observed that both the modulation of LAP phosphorylation levels and increased IL-8 expression in the presence of the PTP1B inhibitor depended on GSK-3 activity. These results indicate that PAF, by rapidly decreasing GSK-3 activation in a PTP1B-dependent manner, would activate a negative feedback mechanism to control the magnitude of response to high concentrations of PAF. Therefore, GSK-3, per se, would be a positive modulator of C/EBPß transcriptional activity and if any negative regulatory sites on C/EBPß would also be phosphorylated, this would have a minor impact on IL-8 transcription. Moreover, given that the inhibition of GSK-3 abrogates the effects of PTP1B inhibition on pThr235 C/EBPß levels, it suggests that Thr235 would be the primary site of GSK-3 phosphorylation, since other activating phosphorylation sites for GSK-3 on C/EBPß depend on phosphorylation of Thr235 and Thr235 is one of the few residues that GSK-3 can phosphorylate without priming phosphorylation [115].

Next, we investigated the pathways by which the PTP1B/SFK complex impaired GSK-3 activity. We focused on Akt, a kinase known to be activated by PAF [116], and given that Src can regulate PI3K-Akt activation, either via direct phosphorylation of PI3K, by increasing its activity, or by facilitating the recruitment of Akt to the membrane subsequent activation [92, 93, 117, 118].

First, we showed that impairing PTP1B activity, either through the PTP1B inhibitor or specific siRNAs, decreased, at all tested times, the phosphorylation of Akt on Ser473, which is one of the markers of Akt activation, suggesting the involvement of PTP1B in this activation. Given that inhibition of SFK by PP2 recapitulated the effects of PTP1B inhibition on pSer473 Akt levels and that a combination of both inhibitors induced a decrease in pSer473Akt levels, similar to those observed with each inhibitor alone, we concluded that PTP1B, via its modulation of SFK activity, up-regulated Akt activation. Moreover, when Akt activation was decreased, either via the inhibition of its upstream activator PI3K by wortmannin or directly by the selective Akt inhibitor MK-2206, a decrease in pSer21/9 GSK-3 levels was observed. This is consistent with results obtained by Nandy et al. who showed that PAF increases pSer9 GSK3ß levels in an Akt-dependent manner in human monocytes [87]. However, the decrease in pSer21/9 GSK-3 levels was observed only at late stimulation times and correlated with a late increase in pTh235 C/EBPß, suggesting that the PI3K-Akt pathway is primarily involved in the regulation of the GSK-3-C/EBP axis, at later stimulation times. Given that the combination of PTP1B inhibitor and wortmannin induced the same phosphorylation pattern of pSer21/9 GSK-3 and pThr235 C/EBP as the PTP1B inhibitor alone, we concluded that the PTP1B/SFK complex could be upstream of this PI3K-Akt-C/EBPß axis. Moreover, given that inhibition of GSK-3 in certain cell types can modulate Akt activation, we examined, by monitoring the pSer473 Akt levels, whether GSK-3 inhibition could modulate Akt activation and thus mediate its effect on C/EBPß phosphorylation and cytokine promoter activation [117]. PAF-induced modulation of pSer473Akt was not influenced by the GSK-3 inhibitor (Additional file 1: Figure S5G and H), so we concluded that that GSK-3 inhibition would not affect Akt activation in our experimental context.

**Fig. 13:**
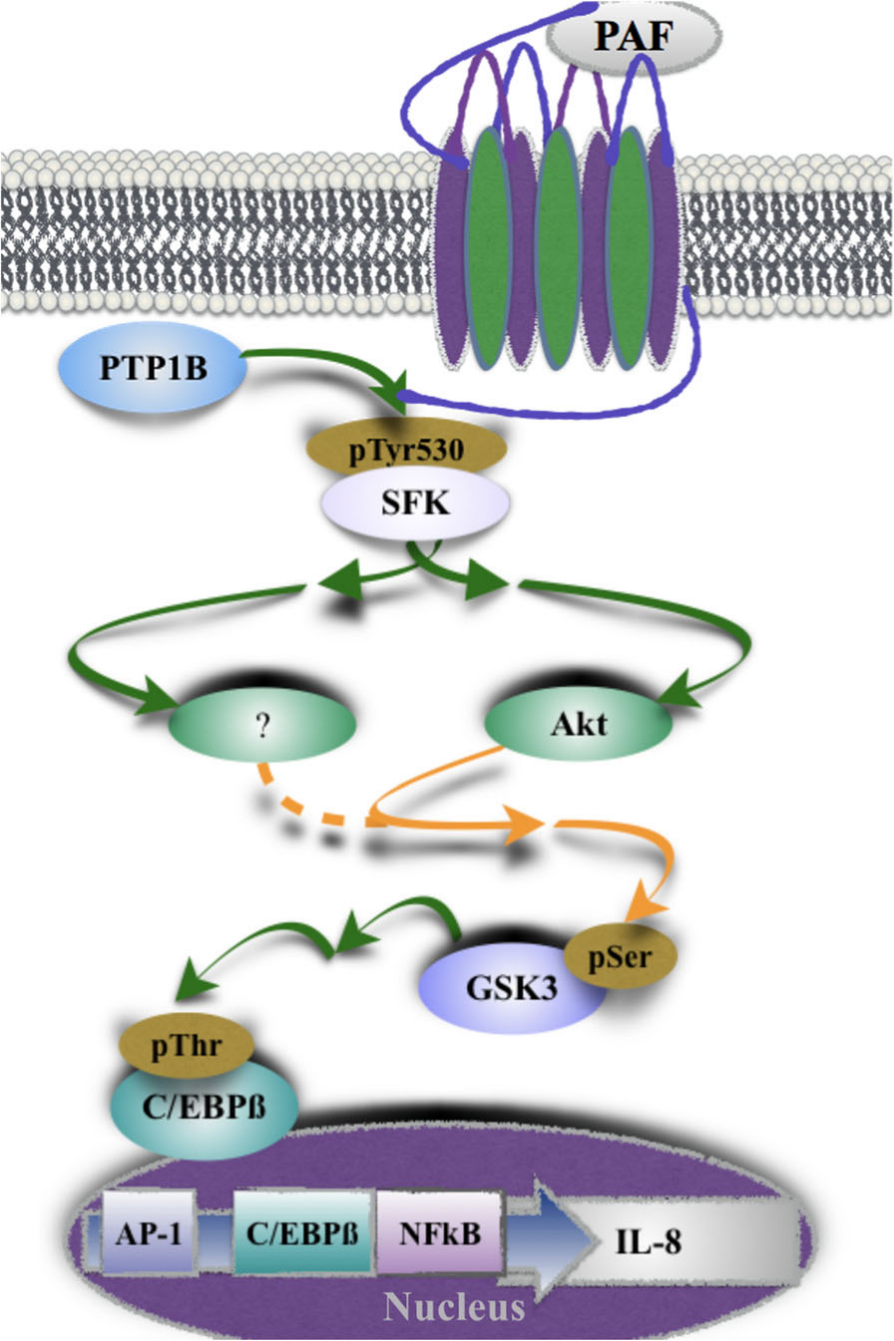
PTP1B modulates IL-8 production via a SFK-Akt-GSK-3-C/EBPß pathway. In iMo-DCs, PAF fine-tunes IL-8 production by activating PTP1B [63] which in turn activates SFK. This PTP1B/SFK signaling pathway up-regulates the phosphorylation of the residues Ser21 of GSK-3α and Ser9 of GSK-3ß, decreasing the capacity of these kinases to phosphorylate their substrates. Whereas the PI3K-Akt pathway mediates the PTP1B/SFK effects on GSK-3 phosphorylation at later stimulation times, the exact mechanism by which the PTP1B/SFK signaling pathway modulates GSK-3 phosphorylation at early stimulation times remains to be identified. These increased GSK-3 phosphorylation levels result in a lower pThr235 C/EBPß and lower IL-8 production

Our results, illustrated in Fig. 13, showing that the PI3K-Akt pathway was involved only at later stimulation times could be surprising given that the PTP1B/SFK complex decreased Akt and GSK-3 phosphorylation levels at all time points. A possible explanation could be that differences in sub-cellular localization or a prerequisite to the formation of a signaling scaffold delayed the action of Akt on GSK-3. For example, arrestins could bring GSK-3 and the Ser/Thr phosphatase PP2A into close proximity, allowing the dephosphorylation of GSK-3 on Ser 21/9 [91, 119]. PP2 and PP1 could also be possible candidates to explain decreased GSK-3 activity at early PAF stimulation times because they also can be activated by SFKs [120–122]. Thus, the mechanisms by which SFKs modulate GSK-3 phosphorylation in the early phases of stimulation remain unclear and will be the object of further investigation.

## Conclusion

Altogether, results reported here suggest that PTP1B can modulate PAF-induced cytokine expression patterns at transcriptional and post-transcriptional levels. More specifically, PTP1B contributes to reducing PAF-induced IL-8 production, both at the mRNA and at protein levels and this, in a C/EBP-dependent but NFκB-independent manner. We found that PAF-stimulated PTP1B activated SFKs and that this PTP1B/SFK signaling pathway downregulated PAF-stimulated IL-8 production via the GSK-3-C/EBPß axis at early stimulation times, where the effector(s) linking SFK to GSK-3 remains to be identified, whereas at late stimulation times this modulation was via the SFK-Akt-GSK-3-C/EBPß pathway.

## Additional file

Additional file 1: Supporting procedure (PDF 4587 kb)

## Abbreviations

AP-1: Activator protein-1
APC: Adenomatous Polyposis Coli Protein
bZIP: basic region leucine zipper
C/EBP: CCAAT-enhancer-binding protein
DC: Dendritic cell
GSK-3: Glycogen synthase kinase
IL-6: Interleukin 6
JNK: c-Jun NH2-terminal kinases
LAP-1: Liver-enriched activator protein
LIP: Liver-enriched inhibitory protein
MMP: Matrix metalloproteinase (MMP)
Mo-DCs: monocyte-derived dendritic cells
PAF: Platelet-activating factor
STAT: Signal transducer and activator of transcription
TGFß: Transforming Growth Factor beta
TNFα: Tumour-Necrosis Factor α
