# Supplementary material for: Regulation of platelet-activating factor-induced interleukin-8 expression by protein tyrosine phosphatase 1B
Source: Cell Commun Signal. 2019 Mar 4;17:21. doi: 10.1186/s12964-019-0334-6 (PMC6399872; doi:10.1186/s12964-019-0334-6)
Supplement: Supplementary file 1 — Supporting procedure (PDF 4587 kb) [file 12964_2019_334_MOESM1_ESM.pdf]

Regulation of platelet-activating factor-induced interleukin-8 expression by protein tyrosine phosphatase 1B

Geneviève Hamel-Côté, Fanny Lapointe, Daniel Gendron, Marek Rola-Pleszczynski and Jana Stankova*

Immunology Division, Department of Pediatrics, Faculty of Medicine and Health Sciences, Université de Sherbrooke, Sherbrooke, QC, Canada

EXPERIMENTAL PROCEDURES

Reagents and chemical products: Anti-FLAG antibodies and paraformaldehyde (PFA) were from Sigma-Aldrich, (Oakville, ON, Canada). Mouse anti-CD1a-FICT or -Phycoerythrin (PE), purified Anti-human IL-8, mouse anti-human CD86 purified IgG1κ, anti-human CD83-PE, -FITC conjugated, mouse Isotype Control IgG1κ, FITC Rat IgG1κ Isotype Control, mouse isotype IgG1κ control antibodies and BD GolgiStop were from BD Pharmingen. Goat anti-mouse IgG-Cy5 antibody was from Jackson Immunoresearch, purchased from Cederlane (Burlington, ON, Canada).

Intracellular cytokine measurement : Mo-DCs were collected on day 7, counted and incubated at 10^6 cells/ml in RPMI+0.2% BSA in 4µL/6mL Golgi-Stop in presence of 10µM PTP1B inhibitor or its vehicle and cells were stimulated with 10nM PAF for 10h or 200ng/mL LPS for 6h with agitation every 30-45min. Cells were centrifuged and fixed with 2% PFA for 15min at room temperature. When necessary, cells were permeabilized with 0.1% saponin for 20min. Non-specific sites and FcR were blocked by incubating cells in 200µl of PBS containing 1% BSA and 150µg/ml human IgG for 15min. Cells were separated into 5 aliquots, 3 of them were stained with antibodies (Abs) against IL-8 and the 2 others received isotype controls. For intracellular staining against IL-8, 0.25µg of mouse anti-IL-8 was added to 2×10^5 cells (final concentration: 6.25ng/µl) and cells were incubated overnight. The cells were then washed 3 times and incubated for 30 min in 40µl of goat anti-mouse Cy5 (dilution: 1:750). After washing 3 times, the secondary Ab was fixed by incubation for 5min in 1% PFA. In anti-IL-8 staining, after 3 washes the cells were incubated for 1h in 40µl of mouse anti-CD1 FITC (dilution 1:100). Cells were rinsed and resuspended in PBS

for subsequent analysis with FACSCAlibur (BD, Biosystems, San José, CA). iMo-DCs were identified by $CD1a^+$ positive staining in the DC gate, determined by FSC and SSC parameters. IL-8 expression levels were determined by GeoMean values.

Luciferase Assays : HEK-PAFR were plated 12 hours before transfection in 24-well plates. Cells were transiently transfected with 200 ng of pcDNA3-hPTP1B and 40ng of luciferase reporter constructs (see plasmid section) per well using 0.75µl of TransIT LT1 transfection reagent according to the manufacturer’s instructions. After 8h in DMEM 5% FBS without puromycin, medium was changed and cells were incubated in DMEM 0.2% BSA for 16h. When mentioned, cells were incubated for 20min with inhibitors (10µM PTP1B inhibitor, 5µM SB 216763) or their vehicle, dimethyl sulfoxide (DMSO). The cells were lysed 6h after stimulation with 100nM PAF. Luciferase activity in lysates was measured as described before (1) using a Sirius luminometer (Berthold detector systems, Montreal, QC, Canada).

RNA Isolation and Real-Time Semi-Quantitative PCR: Mo-DCs were collected on day 7, counted and incubated at 10^6 cells/ml in RPMI+0.2\% BSA for 3 h with agitation every 30-45 min. Next, cells were stimulated with different PAF concentrations for 5 h, then, RNA was obtained using Trizol reagent (Invitrogen, Burlington, ON, Canada) according to the manufacturer's instructions. After quantification, 1.0 μg of RNA was converted to cDNA, QuantiTect Reverse Transcription Kit, according to the manufacturer\textquotesingle s instruction. GAPDH, RPL13a, and IL-8 expression was measured using real-time PCR performed with Syber Green 1 on a Rotor-Gene 3000 (Corbett Research, Kirkland, QC, Canada) as described previously. The following oligonucleotide primer sets were obtained from IDT (Coralville, Ind, USA):
humanGAPDH:
Fwd, 3’-GATGACATCAAGAAGGTGGTGAA-3’
Rvs, 5'-GTCTTACTCCTTGGAGGCCATGT-3'\\
human RPL13a:
Fwd, 5'-GTGCGTCTGAAGCCTACAAG-3'

Rvs 5'-TCTTCTCCACGTTCTTCTCG-3' \\
human IL-8:
Fwd, 5'-TTCTGCAGCTCTGTGTGAAG-3'
Rvs, 5'-AAACTTCTCCACAACCCTCTG-3'\\

Gene expression was normalized with GAPDH mRNA content and differences were calculated with the delta-delta (ΔΔ)Ct method, as in previous studies (2), according to the following formula: (ΔΔCt = [(Ct G.O.I.Ctl - Ct HK.G.Ctl) - (Ct G.O.I.STIM. -Ct HK.G.STIM.)]. Comparison of the expression of each gene between its control and stimulated/siRNA transfected states was determined by ΔΔCt. Results were then transformed into fold variation measurements: fold increase -2ΔΔCt. For IL-8, RPL13a was also used, in parallel to GAPDH, as a housekeeping gene.

Plasmids : The human IL-8 luciferase constructs, pGL3-IL-8wt, pGL3-IL-8-ΔAP-1 (deleted for the AP-1-binding site) or GL3-IL-8-ΔC/EBPß (deleted for AP-1-binding C/EBPß) were kindly provided by Dr Allan R. Brasier from University of Texas Medical Branch (3). The p-NF-κB-luc reporter plasmid was kindly provided by Dr Patrick McDonald (Université de Sherbrooke) (4). The WT PTP1B construction and its dominant negative form (D181A) are described elsewhere (5).

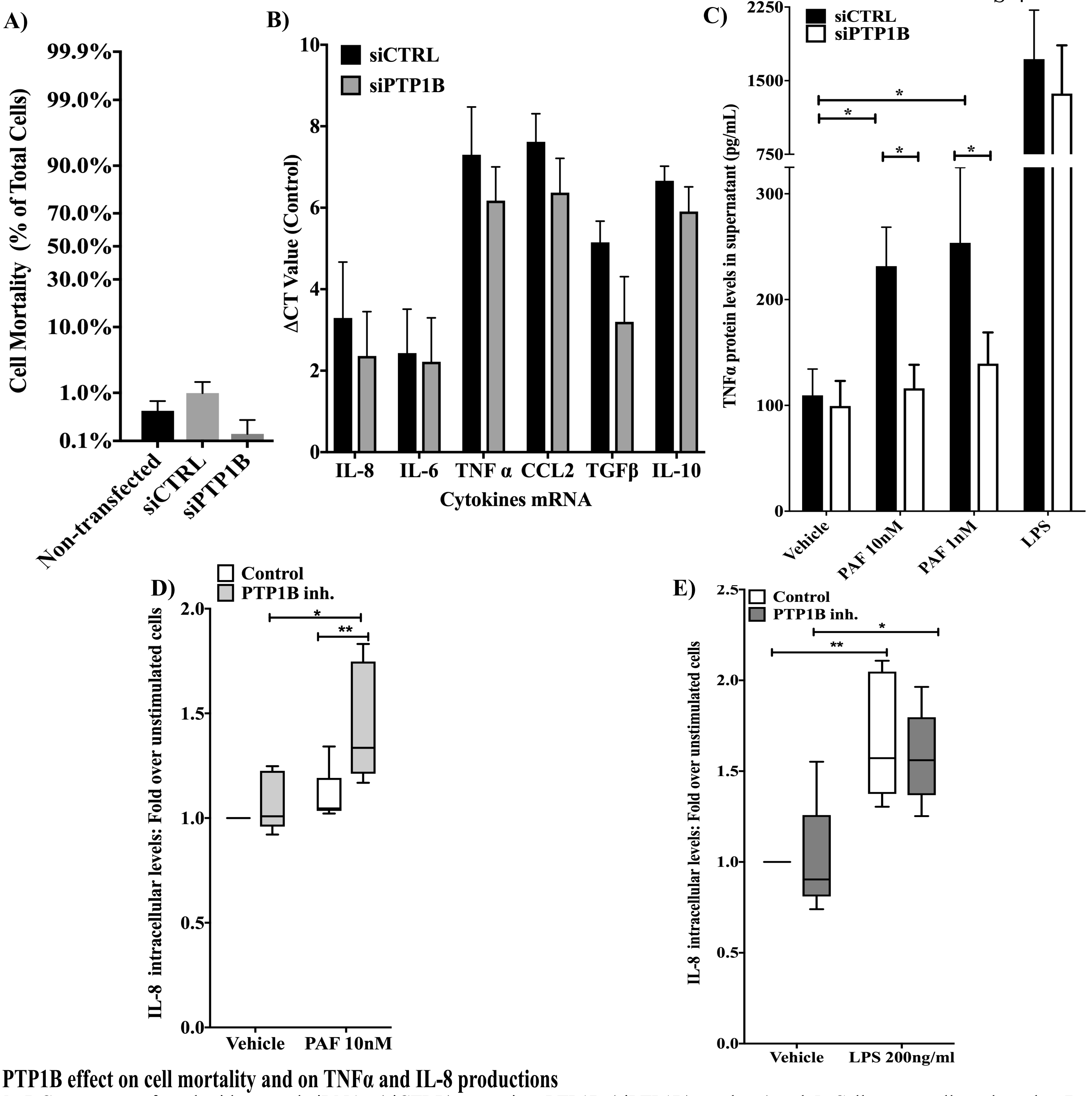

Fig S-1: PTP1B effect on cell mortality and on TNFα and IL-8 productions

A)-C) iMo-DCs were transfected with control siRNAs (siCTRL) or against PTP1B (siPTP1B) on day 4 and 5. Cells were collected on day 7, counted and **A)** cell mortality assed by Trypan Blue Assay. Data are presented as mean±S.E.M of % of Cell Mortality for at least 3 independent experiments. **B)** Cells were lysed in Trizol and RNA was extracted and converted to cDNA. GAPDH, TNFα, IL-6, IL-8, CCL2, TGFß, IL-10 mRNA were quantified by Real-time PCR. Data are presented as mean± S.E.M of mRNA expression calculated by the delta (Δ)Ct method for vehicle-treated cells for 4 independent experiments. **C)** iMo-DCs were resuspended at 10^6 cells/ml in RPMI+0.2% BSA and stimulated with vehicle, 10 nM PAF or 100ng/ml LPS for 24h, with addition of FBS (2% final concentration) after 2h of stimulation. Supernatants were collected and TNFα levels were measured by sandwich ELISA. Data are presented as mean±S.E.M of TNFα levels (pg/ml) in supernatants for at least 3 independent experiments. Significance was established with paired two-way ANOVA with Sidak post-test. *$p<0.05$. **D)& E)** On day 7, iMo-DCs were stimulated in RMPI+0.2% BSA with **D)** 10 nM PAF or its vehicle for 10h or **E)** 200ng/ml LPS or its vehicle for 6h, after a 20 min pre-incubation with the PTP1B inhibitor (10μM) or its vehicle (DMSO). IL-8 intracellular levels were measured in iMo-DCs by flow cytometry. iMo-DCs were identified by CD1a+ positive staining in the DC gate, determined by FSC and SSC parameters. Cells were also tested for the surface expression of CD83, a maturation marker and CD86, a differentiation marker to confirm their immature state. Data are presented in Box-and-whisker (min to max) plot graph of **D)&E)** ratios of IL-8 intracellular levels in stimulated cells determined by the geo mean values, over those in unstimulated, control cells. Significance was established with paired two-way ANOVA with Sidak post-test: *$p<0.05$, ** $p<0.01$

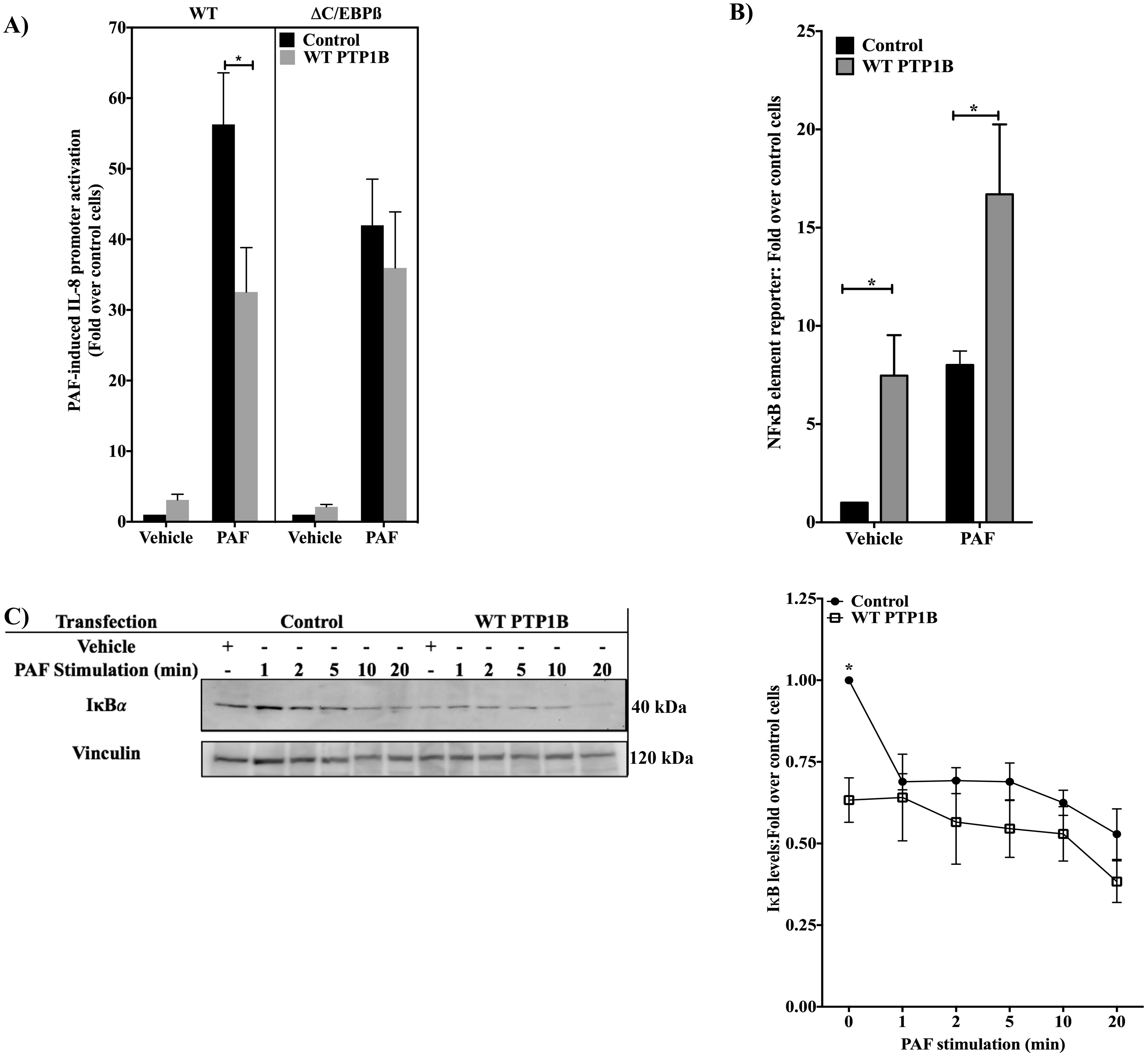

Fig S-2: PTP1B effects on basal IL-8 promoter activity and on PAF-induced NFκB activation

A) HEK-PAFR were co-transfected with WT PTP1B or vector control and pGL3-IL-8 constructs. Cells were starved overnight in DMEM+0.2% BSA and stimulated for 6h with PAF or vehicle and luciferase activity was measured. Data are presented as mean ±S.E.M of fold increase over unstimulated control cells, for at least 4 independent experiments. **B)** HEK-PAFR were co-transfected with PTP1B or vector control and a construct with the NFκB-binding element coupled to the luciferase reporter gene. Cells were starved overnight in DMEM+0.2% BSA and stimulated for 6h with PAF or vehicle and luciferase activity was measured. Data are presented as mean±S.E.M of fold increase over unstimulated control cells of at least 3 experiments. **C)** HEK-PAFR were transfected with PTP1B. Cells were starved overnight in DMEM+0.2% BSA and stimulated for indicated times with PAF 100nM. Representative blots are shown for each condition and compilations of experiments are presented as mean±S.E.M of IkB levels (normalized over vinculin) over normalized values of unstimulated cells, for at least 3 experiments. **A)-C)** Significance was established with paired two-way ANOVA with Sidak post-test: *$p<0.05$.

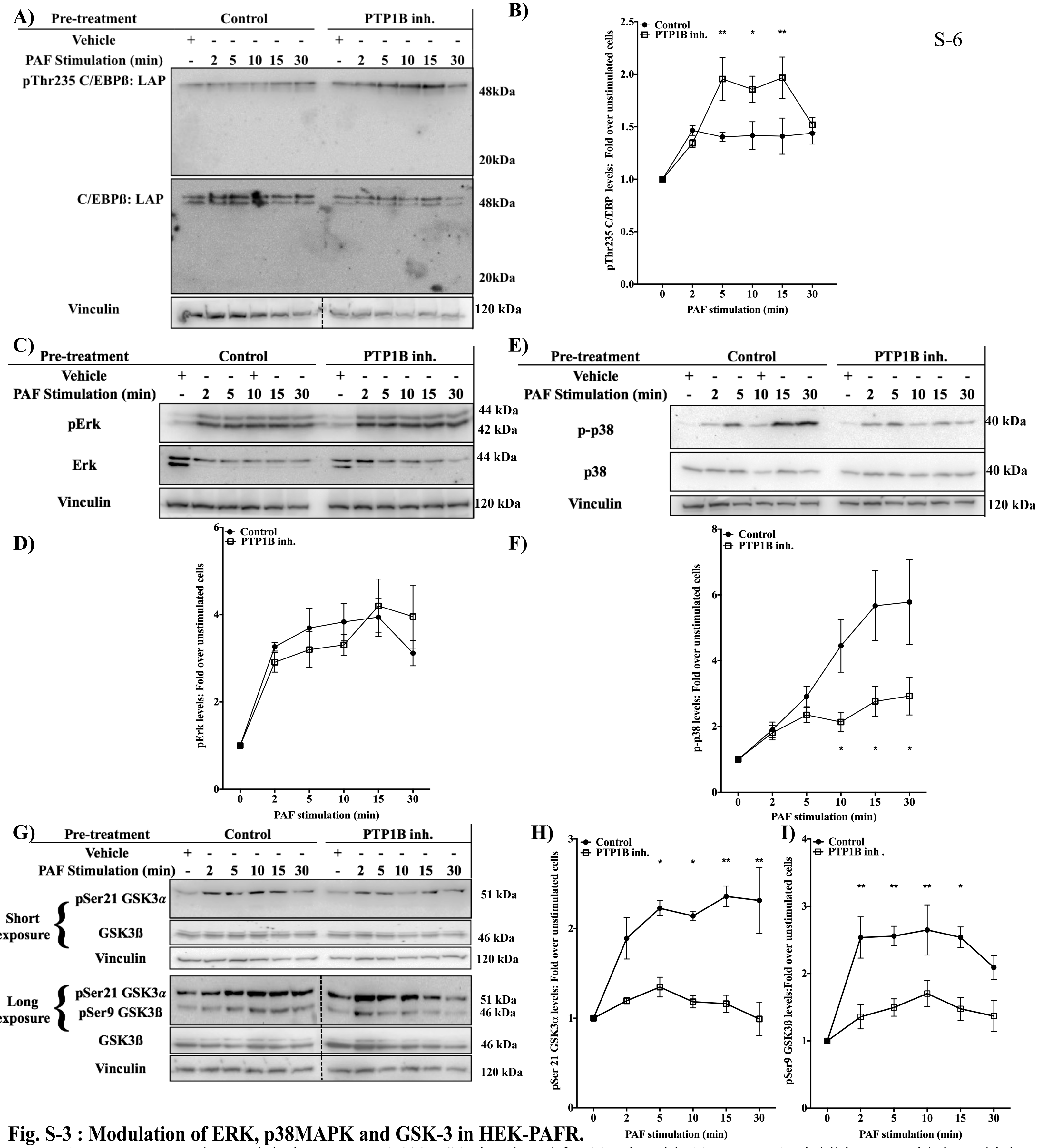

Fig. S-3 : Modulation of ERK, p38MAPK and GSK-3 in HEK-PAFR.
HEK-PAFR were starved overnight in DMEM+0.2% BSA, incubated for 20 min with 10µM PTP1B inhibitor, or with its vehicle and stimulated for indicated times with PAF. Reaction was stopped on ice and cells were collected and lysed. Lysates were separated on SDS-PAGE gels and Western blots were performed with Abs recognizing vinculin and **A)&B)** pThr235 CEBPß and C/EBPß **C)&D)** pTyr204 ERK and ERK, **E)&F)** phospho-(Thr180/Tyr182) p38 and p38. **G)-I)** pSer21/9 GSK3 and GSK3ß. The blots were stripped between re-blotting with total protein antibodies. **G)** A replicate blot was used for long exposure. **A)&G)** The doted lines indicate where the image was slightly rotated for better alignment with the rest of the blot, due to warped migration. Representative blots are shown and compilations of experiments are presented as mean±S.E.M of normalized ratios of phospho-protein levels calculated as described in Materials&Methods, for at least 3 experiments. Significance was established with paired two-way ANOVA with Sidak post-test: *p<0.05

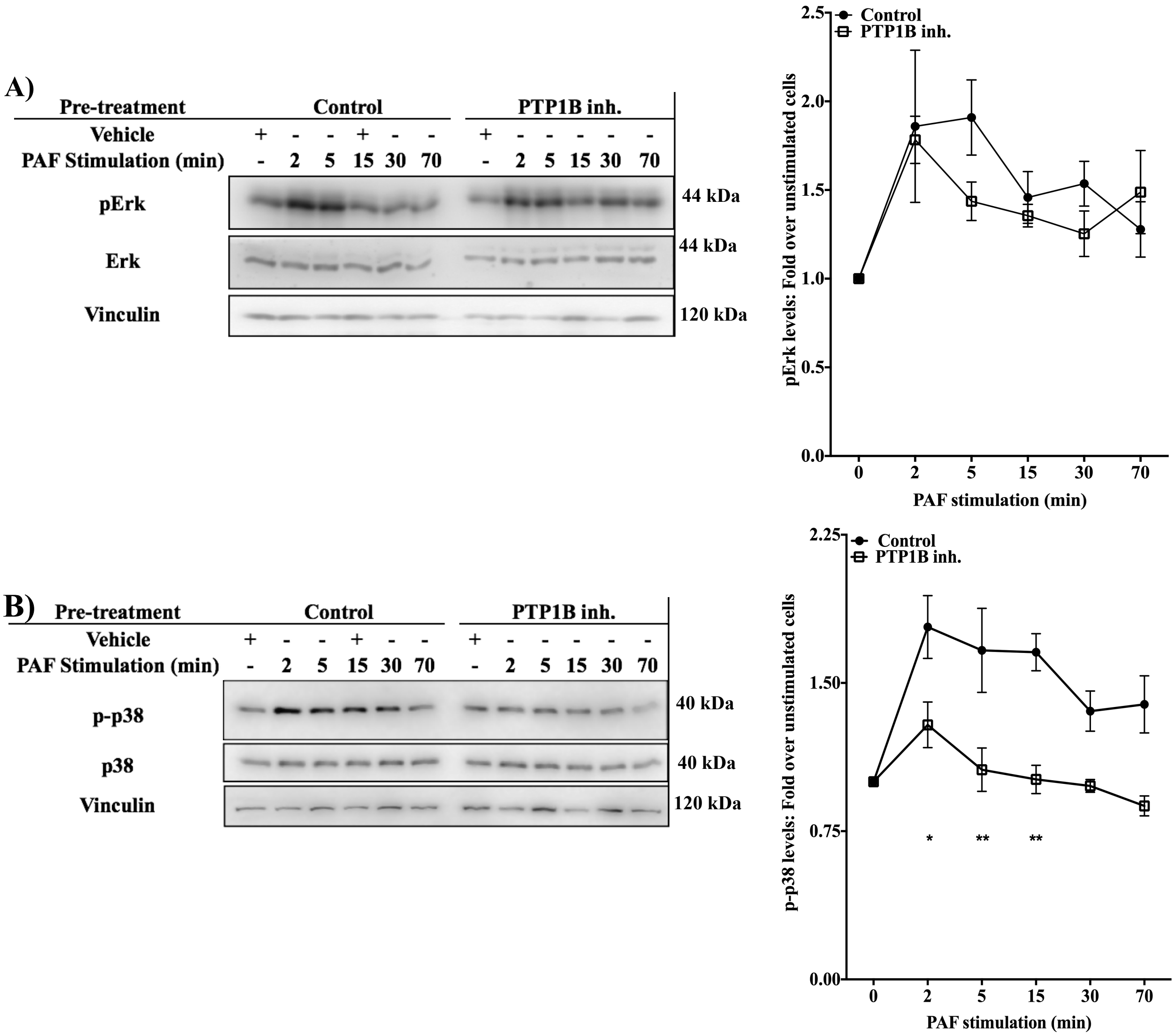

Fig. S-4:PAF-induced ERK and p38MAPK activation are not involved in PTP1B modulation of C/EBPβ phosphorylation.
A)&B) iMo-DCs, collected on day 7, were incubated at 10^6 cells/ml in RPMI+0.2% BSA for 5 h. Then, cells were incubated with 10 µM PTP1B inhibitor before beieng stimulated with 10nM PAF for indicated times and lysed. Lysates were separated on SDS-PAGE gels and Western blots were performed with anti- vinculin **A)** anti-pTyr204 ERK and ERK **B)** anti-phospho-(Thr180/Tyr182) p38MAPK and p38 MAPK Abs. The blots were stripped between re-blotting with indicated antibodies. **B)** The blot presented is the same experiment as in **Fig.4A**, blotted with indicated Abs. Representative blots are shown for each kinase and compilations of at least 3 independent experiments are presented as mean±S.E.M of normalized ratios of phospho-protein levels as described in Materials&Methods. Significance was established with paired two-way ANOVA with Sidak post-test: *$p<0.05$, **$p<0.01$ vs control cells.

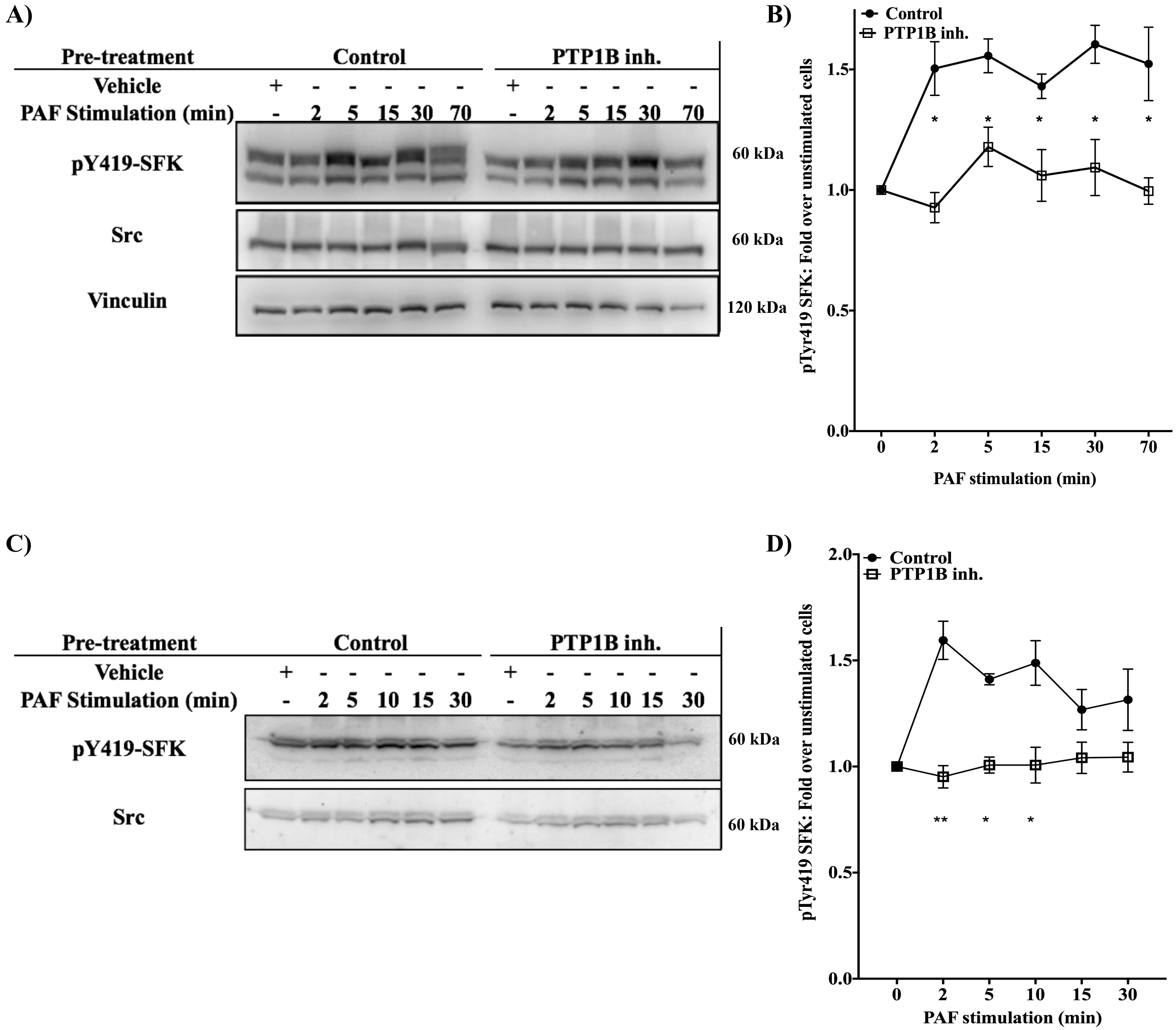

Fig. S-5: PTP1B modulates PAF-induced SFK activation.
A) & B) iMo-DCs were collected on day 7, incubated for 20 min with 10µM PTP1B inhibitor or its vehicle and stimulated for indicated times with PAF (10 nM). **C) & D**) HEK-PAFR were starved overnight in DMEM+0.2% BSA and then incubated for 20 min with 10µM of PTP1B inhibitor or its vehicle. Cells were stimulated for indicated times with PAF (100nM). **A)-D)** Reaction was stopped on ice and cells were collected and lysed. Whole cell lysates were loaded onto SDS-PAGE, transferred to nitrocellulose membrane and blotted overnight with anti-pTyr419 SFK, anti-Src and anti-vinculin Abs . The blots were stripped between re-blotting with indicated antibodies. **A) & C)** Representative blots are shown for each experimental condition and **B) & D)** compilations are presented as mean±S.E.M of normalized ratios of pTyr419 SFK levels calculated as described in Materials&Methods. Significance was established with paired two-way ANOVA with Sidak post-test: *: $p<0.05$; **: $p<0.01$.

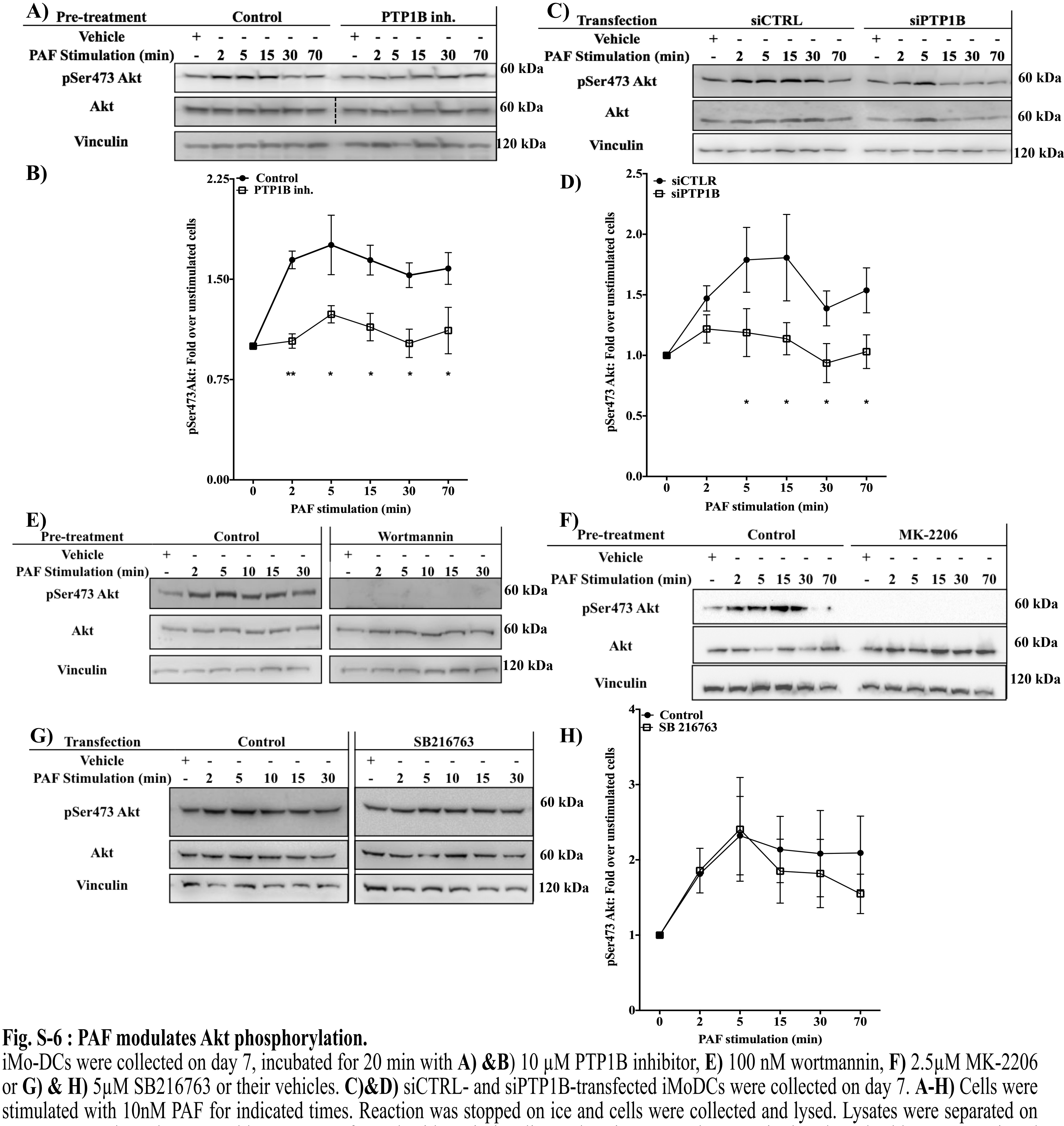

Fig. S-6 : PAF modulates Akt phosphorylation.

iMo-DCs were collected on day 7, incubated for 20 min with **A) &B**) 10 µM PTP1B inhibitor, **E)** 100 nM wortmannin, **F)** 2.5µM MK-2206 or **G) & H)** 5µM SB216763 or their vehicles. **C)&D)** siCTRL- and siPTP1B-transfected iMoDCs were collected on day 7. **A-H)** Cells were stimulated with 10nM PAF for indicated times. Reaction was stopped on ice and cells were collected and lysed. Lysates were separated on SDS-PAGE gels and Western blots were performed with anti-vinculin, and anti-Ser473 Akt or anti-Akt Abs. The blots were stripped between re-blotting with indicated antibodies. **A)** Control and PTP1B inhibitor conditions are on the same blots and the dotted lines indicate where the blot was cut in two in order to use smaller volumes of antibody. **E)&G)** Control and treated cells (wortmanin or SB216764) are shown on separate blots. **E)** The blot presented is the same experiment as in **Fig. 11B)s**, blotted with indicated Abs. **A), C), E)-G)** Representative blots are shown for each condition and **B), D) &H)** compilatioans of experiments are presented as mean±S.E.M of normalized ratios of pSer473 Akt levels calculated as described in Materials&Methods for at least 4 experiments. Significance was established with paired two-way ANOVA with Sidak post-test: *p<0.05, **p<0.01.

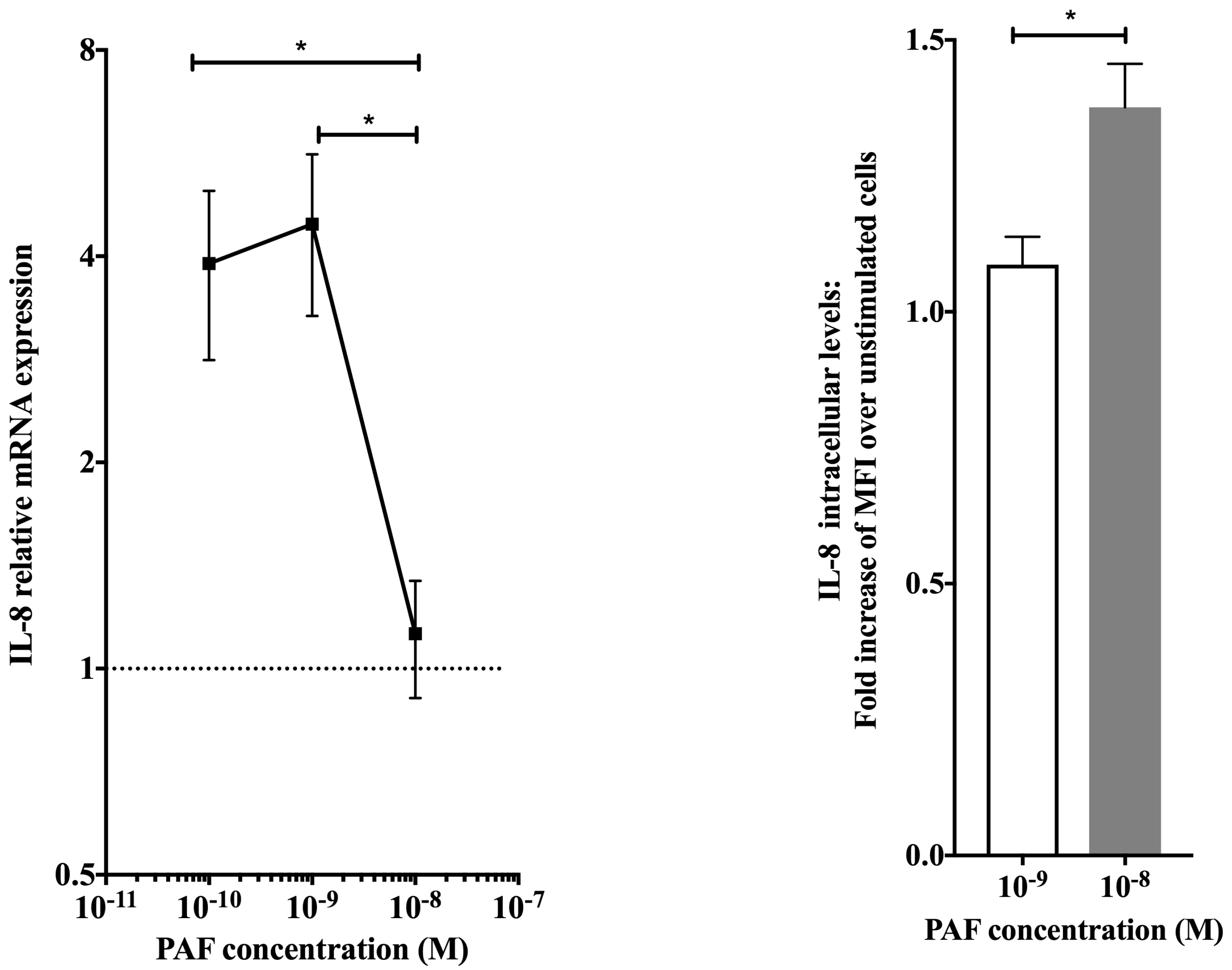

Fig. S-7: PAF induces IL-8 in a concentration-dependent manner A)&B) iMo-DCs were collected on day 7 and stimulated with indicated PAF concentration for **A)** 5h or **B)**10h. **A)** Cells were lysed in Trizol and RNA was extracted and converted to cDNA. GAPDH and RPL13 as housekeeping gene and IL-8 mRNA were quantified by Real-time PCR. Data are presented as mean±S.E.M of mRNA expression calculated by the delta-delta (ΔΔ)Ct method on their unstimulated control for at least 4 independent experiments. Significance was established with Krikall-Wallist test with Dunnett' post-test: *p<0.05 **B)** IL-8 intracellular levels were detected in iMo-DCs by flow cytometry. iMo-DCs were identified by CD1a+ positive staining in the DC gate, determined by FSC and SSC parameters. Cells were also tested for the surface expression of CD83, a maturation marker and CD86, a differentiation marker to confirm their immature state. Data are presented as mean±S.E.M of ratios of IL-8 intracellular levels in stimulated cells determined by the geo mean values, over those in unstimulated, control cells. Significance was established with Wilcoxon test: *p<0.05

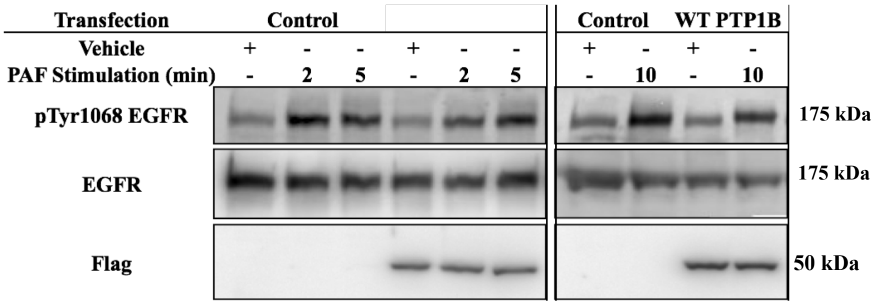

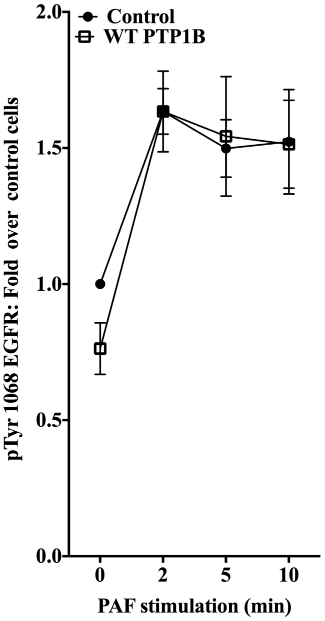

Fig. S-8: Modulation of PAF-induced EGFR activation by PTP1B

HEK-PAFR were transfected with WT PTP1B Flag-tagged construct or control vector, starved overnight in DMEM+0.2% BSA and stimulated for indicated times with PAF 100nM. Reaction was stopped on ice, cells were collected and lysed. Whole cell lysates were loaded onto SDS-PAGE, transferred to nitrocellulose membranes and blotted overnight with Abs recognizing Flag, pTyr1068 EGFR and EGFR. The blots were stripped between re-blotting with Abs recognizing EGFR. Data are presented as mean±S.E.M of normalized ratios of phospho-protein levels calculated as described in Materials&Methods, for 4 experiments. Significance was established with paired two-way ANOVA with Sidak post-test.

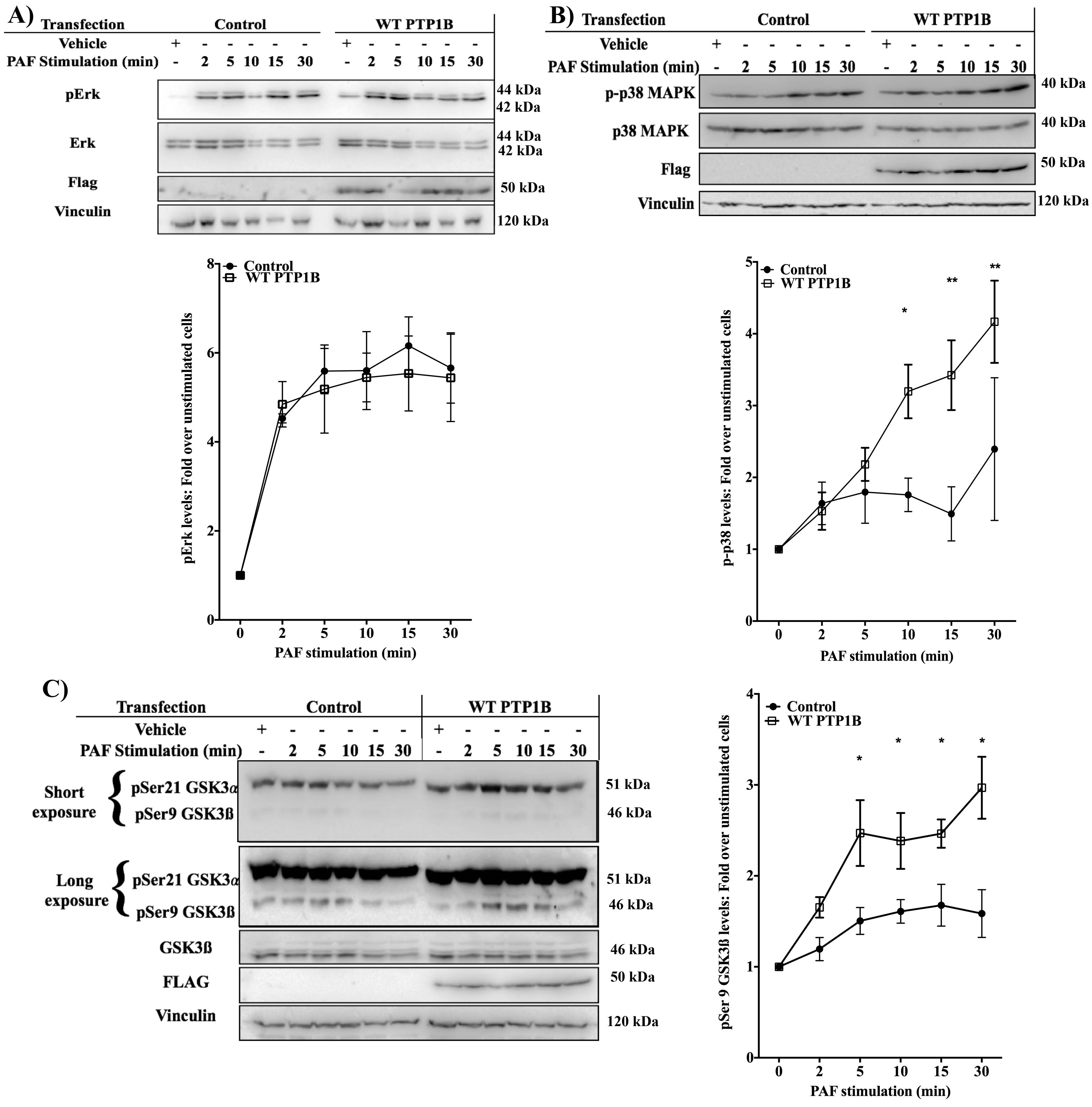

Fig.S-9 : Modulation of ERK, p38MAPK and GSK3 in WT PTP1B-transfected HEK-PAFR.
HEK-PAFR were transfected with WT PTP1B, starved overnight in DMEM+0.2% BSA and stimulated for indicated times with PAF 100 nM. Reaction was stopped on ice and cells were collected and lysed. Lysates were separated on SDS-PAGE gels and Western blots were performed withAbs recognizing vinculin, Flag and **A)** pTyr204 ERK and ERK, **B)** phospho-(Thr180/Tyr182) p38 MAPK and p38 MAPK, **C)** pSer21/9 GSK-3 and GSK-3ß. The blots were stripped between re-blotting with indicated antibodies. **C)** A replicate blot was used for anti-FLAG Abs, same samples had been used for blots in **S-9B**). Representative blots are shown and compilations of experiments are presented as mean±S.E.M of normalized ratios of phospho-protein levels calculated as described in Materials&Methods, for at least 3 experiments. Significance was established with paired two-way ANOVA with Sidak post-test: *$p<0.05$.

REFERENCES

1. Thompson, C., Cloutier, A., Bossé, Y., Thivierge, M., Gouill, C. L., Larivée, P., McDonald, P. P., Stankova, J., and Rola-Pleszczynski, M. (2006) CysLT1 receptor engagement induces activator protein-1- and NF-kappaB-dependent IL-8 expression. *Am. J. Respir. Cell Mol. Biol.* **35**, 697–704
2. Drolet, A.-M., Thivierge, M., Turcotte, S., Hanna, D., Maynard, B., Stankovà, J., and Rola-Pleszczynski, M. (2011) Platelet-activating factor induces Th17 cell differentiation. *Mediat. Inflamm.* **2011**, 913802, 10.1155/2011/913802
3. Vlahopoulos, S., Boldogh, I., Casola, A., and Brasier, A. R. (1999) Nuclear Factor-κB Dependent Induction of Interleukin-8 Gene Expression by Tumor Necrosis Factor α: Evidence for an Antioxidant Sensitive Activating Pathway Distinct From Nuclear Translocation. *Blood*. **94**, 1878–1889
4. Ear, T., and McDonald, P. P. (2008) Cytokine generation, promoter activation, and oxidant-independent NF-κB activation in a transfectable human neutrophilic cellular model. *BMC Immunol.* **9**, 14
5. Hamel-Côté, G., Gendron, D., Rola-Pleszczynski, M., and Stankova, J. (2017) Regulation of platelet-activating factor-mediated protein tyrosine phosphatase 1B activation by a Janus kinase 2/calpain pathway. *PloS One*. **12**, e0180336
